# Exploring CVD Method for Synthesizing Carbon–Carbon Composites as Materials to Contact with Nerve Tissue

**DOI:** 10.3390/jfb14090443

**Published:** 2023-08-28

**Authors:** Aneta Fraczek-Szczypta, Natalia Kondracka, Marcel Zambrzycki, Maciej Gubernat, Pawel Czaja, Miroslawa Pawlyta, Piotr Jelen, Ryszard Wielowski, Danuta Jantas

**Affiliations:** 1 Faculty of Materials Science and Ceramics, AGH University of Science and Technology in Krakow, Mickiewicza 30 Av., 30-059 Krakow, Poland; zambrzycki@agh.edu.pl (M.Z.); maciej.gubernat@agh.edu.pl (M.G.); pjelen@agh.edu.pl (P.J.); rwielows@agh.edu.pl (R.W.); 2 Faculty of Electrical Engineering, Automatics, Computer Science and Biomedical Engineering, AGH University of Science and Technology in Krakow, Mickiewicza 30 Av., 30-059 Krakow, Poland; n.kondracka98@gmail.com; 3 Institute of Metallurgy and Materials Science, Polish Academy of Science, Reymonta 25 St., 30-059 Krakow, Poland; czaja.p@imim.pl; 4 Materials Research Laboratory, Faculty of Mechanical Engineering, Silesian University of Technology, Akademicka 2A Str., 44-100 Gliwice, Poland; mpawlyta@polsl.pl; 5 Department of Experimental Neuroendocrinology, Maj Institute of Pharmacology, Polish Academy of Sciences, Smetna 12 Str., 31-343 Krakow, Poland; jantas@if-pan.krakow.pl

**Keywords:** carbon fibers, pyrolytic carbon, C/C composites, materials for nerve stimulation, CVD method

## Abstract

The main purpose of these studies was to obtain carbon–carbon composites with a core built of carbon fibers and a matrix in the form of pyrolytic carbon (PyC), obtained by using the chemical vapor deposition (CVD) method with direct electrical heating of a bundle of carbon fibers as a potential electrode material for nerve tissue stimulation. The methods used for the synthesis of PyC proposed in this paper allow us, with the appropriate selection of parameters, to obtain reproducible composites in the form of rods with diameters of about 300 µm in 120 s (CF_PyC_120). To evaluate the materials, various methods such as scanning electron microscopy (SEM), scanning transmission electron microscope (STEM), high-resolution transmission electron microscopy (HRTEM), selected area electron diffraction (SAED), Raman spectroscopy, X-ray photoelectron spectroscopy (XPS), and tensiometer techniques were used to study their microstructural, structural, chemical composition, surface morphology, and surface wettability. Assessing their applicability for contact with nervous tissue cells, the evaluation of cytotoxicity and biocompatibility using the SH-SY5Y human neuroblastoma cell line was performed. Viability and cytotoxicity tests (WST-1 and LDH release) along with cell morphology examination demonstrated that the CF_PyC_120 composites showed high biocompatibility compared to the reference sample (Pt wire), and the best adhesion of cells to the surface among all tested materials.

## 1. Introduction

The nervous system acts as a complex management center for the human body, relying on multiple factors like genetics, external influences, and aging for proper functioning. Neurodegenerative diseases, including Alzheimer’s, ALS, Huntington’s, and Parkinson’s, are examples of nervous system malfunctions [1,2,3]. These conditions lead to a gradual loss of nerve cells in different brain regions, causing nervous system dysfunction [3].

Treating neurodegenerative diseases involves pharmacological methods as well as deep brain stimulation (DBS) [4,5,6]. DBS utilizes implanted electrodes in specific brain areas, customized to the disease type, along with a neurostimulator. This system works to inhibit abnormal neuron activity [5,7]. DBS has been employed for many years to treat severe movement disorders like Parkinson’s, tremors, dystonias, and chorea, as well as central pain syndromes, epilepsy, and certain mental disorders [5,7]. The most commonly used materials for DBS electrodes are various metals and metal alloys, like platinum or platinum–iridium (Pt-Ir) [8]. These materials are preferred for their ability to stimulate neurons effectively and their biocompatibility.

Current neuromodulation techniques for DBS have several major drawbacks. These include the large size of the implanted devices, with electrode diameters exceeding 1 mm. There is also a lack of feedback monitoring of brain electrical activity, high demand for electrical current, and the risk of brain hemorrhage due to the numerous microelectrodes passing through the brain [9,10]. Another significant issue with DBS electrodes is the formation of glial scar tissue around them. This leads to an increase in electrical resistance between the electrodes and nerve tissue, requiring a higher voltage and current for nerve stimulation [11,12,13,14]. As a result, the battery drains faster, shortening the electrodes’ lifespan. Additionally, glial scar tissue can cause brain trauma due to long-term inflammation [15]. Furthermore, the stiffness of the electrodes, typically greater than the surrounding tissue, can result in tissue detachment [16].

Challenges in electrode use and design include the size of the electrodes, their diameter-to-length ratio, and achieving miniaturization [9,17]. Optimal electrode size influences the quality and effectiveness of brain stimulation, while the diameter-to-length ratio impacts the area affected by the stimulation. Carbon materials, due to the variety of forms, allotropes, and the resulting very different properties, are often considered potential materials for electrodes or substrates for stimulation and regeneration of nervous tissue. Among these materials, we can mention carbon nanomaterials such as nanotubes (CNT), graphene and its derivatives, carbon nanofibers (CNF), and finally, carbon fibers (CF) and their composites [18,19,20,21,22,23,24,25,26]. Especially, carbon fibers (CF) with diameters of single micrometers are potential materials for electrodes and microelectrodes for brain stimulation. 

Carbon fibers with diameters ranging from 4 to 10 μm allow precise control when combined into cylindrical electrodes [27]. Their various thicknesses, stiffnesses, and surface profiles make customization for specific tissues possible [28]. The superior properties of polyacrylonitrile-based carbon fibers (PAN-based carbon fibers), including tensile strength, thermal and chemical resistance, and electrical conductivity, surpass other options. The carbon surface can be easily modified for desired outcomes, impacting biocompatibility and reducing scar tissue formation [29]. Carbon fiber electrodes, smaller than conventional metal wire electrodes, show better capabilities for chronic neural recording and cause less tissue damage [30]. They also offer promise for magnetic resonance (MR) compatibility, ensuring safety during MR acquisitions [31,32]. Adverse implant interactions, such as heating, forces, induction, and MR artifacts, are important considerations [26]. Precise electrode positioning is vital for therapy effectiveness, using imaging methods like magnetic resonance imaging (MRI) during surgery [10]. Post-procedure, the electrode location is closely monitored [8]. Hence, MR compatibility is crucial to avoid artifacts and heating during imaging. 

Carbon fiber shows promise as an electrode material with a wide range of research and application possibilities [25,26]. It can be used for neurotransmitter detection, monitoring, and recording signals from the nervous system [26,30,33,34]. Microelectrodes based on carbon fibers outperform conventional electrodes, ensuring stable neural recording without signal deterioration over time [30]. Graphitized fibers (GF) have also been tested for neuronal stimulation, exhibiting low impedance, a wide electrochemical window, and stability for 24 days [35]. In rats with Parkinson’s disease, GF showed the ability to stimulate cells and alleviate symptoms.

While carbon fibers offer numerous advantages for nervous tissue applications, they are highly mechanically fragile [36]. Their limited insertion depth in cortex regions, less than 2 mm, is due to the individual fibers’ insufficient stiffness to penetrate deeper brain regions despite their high mechanical properties [30,36]. Improving the durability and stiffness of individual fibers is crucial. One method reported in the literature is the application of DLC-based carbon coatings or diamond, obtained through the CVD method [25,29,36]. Pyrolytic carbon (PyC), another option similar to DLC, can also enhance carbon fibers’ stiffness and usability. Low-temperature pyrolytic carbon is a well-known and highly biocompatible material used in medicine [37].

Pyrolytic carbon is usually obtained in the form of layers of varying thicknesses on various substrates [38]. Depending on their designation, they can perform various functions, e.g., they may improve the biocompatibility of modified materials in contact with blood or improve the wear resistance of friction elements in joint endoprostheses [39,40,41]. Among the materials available for mechanical heart valve prostheses, pyrolytic carbon has the best combination of blood compatibility, physical and mechanical properties, and durability [37,39]. Pyrolytic carbons can be obtained by, among other methods, CVD, where hydrocarbons, e.g., methane or propane are used as carbon-containing compounds [38,42]. What is interesting is that pyrolytic carbon can have a variety of structures, such as smooth and dark laminar or isotropic [43]. The structure of the pyrolytic carbon is controlled by the gas flow rate, hydrocarbon species, temperature, and bed surface area. Pyrolytic carbon can also be obtained as a matrix in carbon-carbon (C/C) composites using the chemical vapor infiltration (CVI) method belonging to the family of CVD methods [44,45]. In contrast to CVD, in CVI the deposition takes place within porous preforms usually made of fibers. Thus, the gaseous precursor penetrates through the preform pores and undergoes a chemical reaction thereby depositing in the pores [46]. In this way, the matrix material grows into a fibrous porous structure (preform) in a continuous layer-by-layer way, thus forming the composite matrix [37]. 

One significant drawback of obtaining pyrolytic carbon through CVD methods is the long synthesis time, which can extend to several thousand hours, depending on the sample’s size. To address this, the researchers in this work used the CVD method with direct heating of the sample to prepare C/C composites. This method is very poorly covered in the literature and so far only one article has described this technique [47]. They created a dedicated system for synthesizing rod-shaped composites, using a fiber bundle as the core and pyrolytic carbon as the matrix. This method allows for the rapid synthesis of pyrolytic carbon (several minutes) to obtain a composite with a controlled diameter and length.

While pyrolytic carbon is known in cardiac surgery and orthopedics, it represents a novelty in the field of neurosurgery. Also, to the best of our knowledge, the concept of C/C composites in the area of stimulation of nerve tissue cells has never been previously studied and is yet to appear in the literature. In addition, the CVD method with direct electrical heating of a bundle of carbon fibers proposed in this work is also a very interesting and innovative tool for the synthesis of C/C composites.

The main goal of this pioneering study on carbon electrodes in the form of rods for stimulating nerve tissue cells was to optimize the electrode production process, assess the microstructural and structural properties of the materials obtained, and evaluate their initial biocompatibility in vitro with SH-SY5Y human neuroblastoma cells. The optimization focused on the quantity of fibers in the composite, affecting the electrode diameter, and the synthesis time for pyrolytic carbon (PyC). Scanning microscopy examinations characterized the microstructure, fiber bundle filling with pyrocarbon, and porosity. The functional properties of the composites were influenced by the structure of pyrolytic carbon, and evaluated using high-resolution transmission microscopy (HRTEM), selected area electron diffraction (SAED), and Raman spectroscopy. Surface chemistry and wettability were measured using the XPS technique and a tensiometer, respectively. To utilize these C/C composite-based electrodes in the future, cytotoxicity and cell viability assessments were essential. The SH-SY5Y cell line was chosen for this purpose, as it is commonly used in neurodegenerative disorder models and neurological research experiments [48,49,50,51]. This study marks the first step towards further research on carbon electrodes based on carbon fibers and PyC for stimulating nerve tissue cells.

## 2. Materials and Methods

### 2.1. Materials

#### 2.1.1. Carbon Fibers

**Figure 1:**
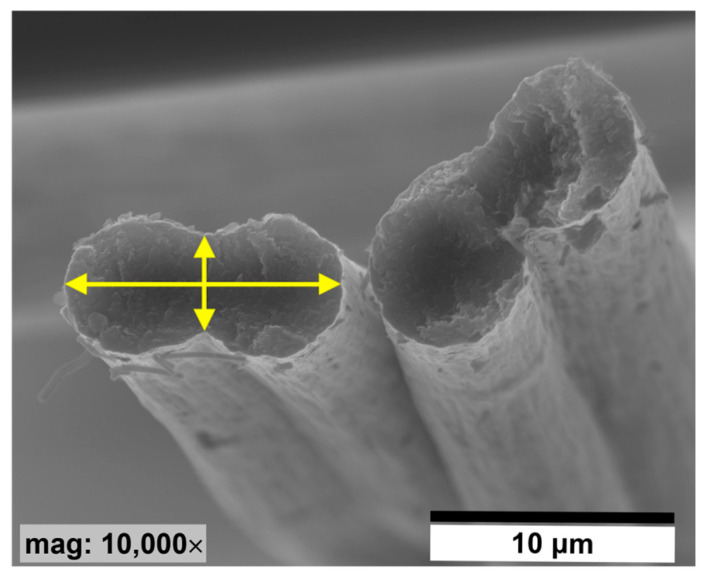
SEM image of dog-bone-shaped CFs cross-sectional.

In order to obtain carbon-carbon (C/C) composites, high-modulus carbon fibers (CF) obtained from a polyacrylonitrile (PAN) precursor by Celanese Co., USA were used. The basic parameters of the CF from the datasheet are: the number of filaments in a bundle of 250, tensile strength of 1.8 GPa, and Young’s modulus of 500 GPa. The carbon fibers used for the study have a dog-bone-like shape (Figure 1). The longer diameter of the fiber is about 12.42 ± 0.79 µm, and the shorter diameter is about 4.38 ± 0.41 µm (Figure 1, arrows). The main factor determining the selection of this type of fiber was the initial small number of carbon fibers in the bundle and the low specific resistance of these materials. Additionally, it was advisable to use CF based on the polyacrylonitrile precursor, and not based on pitch, although the latter have higher electrical conductivity. The low number of fibers in the bundles facilitates their formation and separation in order to obtain C/C composites with a small diameter. 

#### 2.1.2. CVD Method with Direct Electrical Heating of Carbon Fibers

**Figure 2:**
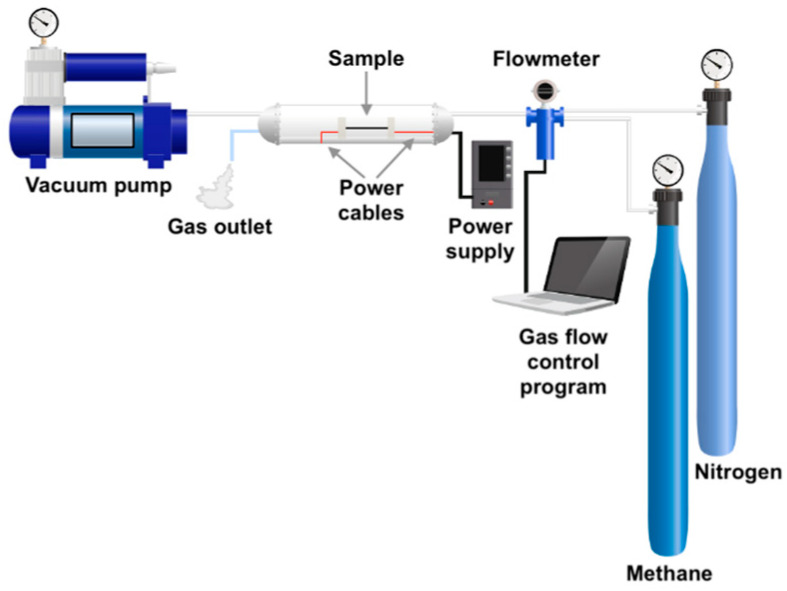
Scheme of the system for the synthesis of pyrolytic carbon using the CVD method with direct electrical heating of the sample.

Carbon–carbon (C/C) composites were obtained as a result of pyrolysis of a gaseous precursor (methane) in order to obtain pyrolytic carbon (PyC). The synthesis method is an upgraded version of the chemical vapor deposition (CVD) method using direct electric heating of a bundle of carbon fibers. This method allows for very quick heating (single seconds) of a bundle of fibers to a predetermined temperature thanks to the use of a DC generator in the reactor into which the carbonaceous gas is introduced directly. Direct heating of fibers causes the gas pyrolysis process to take place in the hottest zone, in this case directly on the surface of the fibers or a short distance from their surface, which makes the pyrolytic yield much higher than in the case of the classic CVD method or the CVI method. The latter is very effective in the process of obtaining C/C composites, but it requires many densification cycles, which makes it long-lasting and expensive. In addition, the method proposed in this work allows one to obtain a large amount of pyrocarbon in a short time, i.e., up to 0.5–3 minutes, depending on the amount of carbonaceous gas introduced. The device was manufactured specifically for the project and the production of C/C composites in the form of thin rods and is called CFCPP-1100, carbon fiber pyrolytic carbon coating by Fine Instruments, Poland. The circuit diagram is shown in the image below (Figure 2). The system consists of a quartz glass reactor, a system for assembling fiber samples (two pairs of graphite elements between which a bundle of carbon fibers was introduced and through which the current was passed), a vacuum pump, a DC generator, a system for gas supply and extraction, a flow counter, gas cylinders and a pyrometer to control the temperature of the fiber bundle.

**Figure 3:**
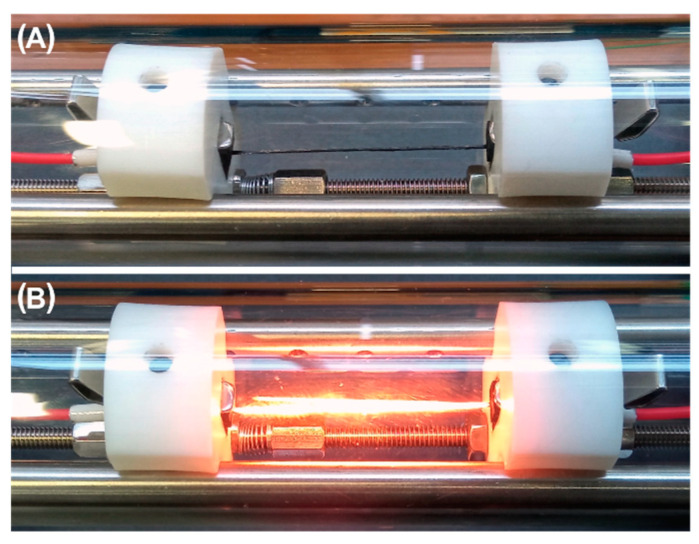
Bundle of fibers before (**A**) and during (**B**) synthesis.

For the purpose of synthesis, a bundle of carbon fibers with the appropriate number of fibers in the bundle was placed between graphite elements and heated to the set temperature in the range of 1100–1200 °C. The image below shows a bundle of fibers before and during heating (Figure 3A,B). Prior to the CVD synthesis process, a vacuum is created to better remove air from the reactor as well as from the spaces between the individual fibers in the bundle. Then, an inert gas (N_2_) is introduced into the reactor and the vacuum is removed. The reactor is flushed with N_2_ for 60 seconds. After this time, a carbonaceous gas (CH_4_) is introduced into the reactor while the flow of N_2_ is maintained. The amount of CH_4_ introduced into the reactor is 2 L/h, while the amount of N_2_ is 10 L/h. The gas injection time varies, i.e., from 30 s to 180 s. In the next stage, the sample is kept at the synthesis temperature for 30 s in an inert atmosphere, without the flow of carbonaceous gas. Finally, the system is cooled to room temperature (RT) by reducing the voltage on the DC power supply to zero. The total time needed to carry out the entire synthesis from the introduction of the sample into the reactor to its removal after synthesis is a maximum of 6–7 min.

Using this method, the following 4 types of C/C composites were produced:

- CF_PyC30—rod-shaped C/C composite based on carbon fiber and PyC obtained after 30 s of synthesis.
- CF_PyC60—rod-shaped C/C composite based on carbon fiber and PyC obtained after 60 s of synthesis.
- CF_PyC120—rod-shaped C/C composite based on carbon fiber and PyC obtained after 120 s of synthesis.
- CF_PyC180—rod-shaped C/C composite based on carbon fiber and PyC obtained after 180 s of synthesis.
- CF—bundle of carbon fibers.

Each bundle of fibers synthesized by CVD had between 200 to 220 individual fibers per bundle. The number of fibers was dictated by the need to obtain a rod-shaped C/C composite with a diameter of less than 1 mm and good handiness.

### 2.2. Methods

#### 2.2.1. SEM and Digital Microscope

The evaluation of the microstructure and morphology of carbon fibers and C/C composites was performed using Nova NanoSEM 200 (FEI Europe Company, Eindhoven, The Netherlands) scanning electron microscope and Thermo Fisher Scientific (Waltham, MA, USA) SCIOS II Dual Beam scanning electron microscopes (SEM). The acceleration voltage was 10 kV. The SEM images were also used to evaluate the diameter of carbon fibers and C/C composites and also to evaluate diameters of ball-like protuberances characteristic for pyrocarbon after using ImageJ v1.53e software, developed at the National Institutes of Health and the Laboratory for Optical and Computational Instrumentation (LOCI, University of Wisconsin), USA, public domain. In total, 70 protuberance measurements were made on the surface of PyC. Moreover, the porosity of all C/C composites was established based on SEM microphotographs using ImageJ software. For porosity thresholding of SEM images was performed. Thresholding is a type of image segmentation where the pixels of an image are changed to make the image easier to analyze. In thresholding, the images are converted from color or grayscale into a binary image, i.e., one that is simply black and white.

A digital microscope (VHX-900F, Keyence Co., Mechelen, Belgium) was also used to analyze the surface morphology of the samples. Equipped with two lenses, a standard lens, and a Z500T lens, it allows images at 20×–200× and 500×–5000× magnification. The working distance is 4.4 mm.

#### 2.2.2. TEM and HRTEM

Detailed characterization of microstructure and structure was performed with a Thermo Fisher Scientific (Waltham, MA, USA) Titan Themis Cs corrected 200 kV XFEG transmission electron microscope (TEM). Thin foils for TEM inspection were cut out with a focused ion beam (FIB) technique employing Thermo Fisher Scientific SCIOS II Dual Beam scanning electron microscopes. Thin lamellas for TEM inspection were cut out from cross-sections of carbon rods, from the boundary separating the matrix and the inner rod. The voltage applied was 30 kV, while the current was initially set to 30 nA during regular cross-section milling, and subsequently it was reduced down to 3 nA. Once the lamellae were cut out it was transferred onto a copper grid with a lift-out omniprobe system. The lamella was welded to the grid with Pt and further thinned with the beam current gradually decreasing from 3 nA to 50 pA. Then it was transferred onto a TEM holder and examined.

#### 2.2.3. Selected Area Electron Diffraction (SAED)

The obtained SAED images were used to quantify the degree of preferred orientation, the so-called orientation angle, OA. The procedure included the following steps:

- Determining the position of the center of the diffraction pattern and the radius of the diffraction ring with indices (002);
- Determining (using a self-developed script in Python) the profile of intensity changes along the perimeter of a circle with a predetermined center and radius (values read in 0.2 degree steps);
- Fitting two Gaussian curves to the obtained profile, the maximum values of which occur at points of the circle (located on opposite sides) with the highest intensity, and determining the half-width FWHM of these curves [52,53];
- Determination of their average value, equal to orientation angle OA.

#### 2.2.4. Raman Spectroscopy

Raman spectroscopy measurements were performed using a WITec Alpha 300 M + apparatus with a 600 g/mm grating, a 488 nm diode laser, and a 50× lens. A total of 10 accumulations with 20 s integration times were recorded for each point in the line measurement. Fityk 0.8.0 software was used for the spectra analysis. Spectral deconvolution was performed using the Voigt function [54]. It allowed us to distinguish characteristic bands corresponding to vibrations of carbon structures in samples. The I_D_/I_G_ ratio was determined from the total intensities of the D and G bands as a coefficient describing the degree of crystallinity of the carbons. Additionally, the size of the L_a_ crystallite was also determined using the Cançado equation [55]:

(1) $$L_{a}=\left(2.4\times 10^{-10}\right)\times {\;\lambda }^{4}\times {\left(\frac{I_{D}}{I_{G}}\right)}^{-1}$$

where L_a_ is a crystallite size (nm), λ is the radiation wavelength (nm) and I_D_/I_G_ are the intensity of Raman D and G bands.

#### 2.2.5. XPS

The surface chemistry of the samples was determined using the X-ray photoelectron spectroscopy (XPS) spectroscope PHI VersaProbe II Scanning XPS system working with a monochromatic Al source—line Kα (1486.6 eV). Energy pass was set to 117.50 eV for the survey scan and 46.95 eV for core-level spectra. The charge compensation was ensured with a dual beam of 7 eV Ar^+^ ions and 1 eV electrons. The operating pressure in the analytical chamber was < 3 × 10^−9^ mbar, and the area of focus of X-rays was 100 µm. Estimated depth of analytical information was about 5 nm. The shift of energy due to the charging effects was calibrated assuming binding energy of C1s line = 285 eV. Fitting of core-level spectra and background subtraction using the Shirley method were performed using PHI MultiPak software (v.9.9.2). 

#### 2.2.6. Contact Angle Measurement

The contact angle 20 µm, measuring range: 0–180° and resolution 0.01°. The proposed method of assessing wettability is a dynamic method in which the so-called advancing angle (θ_Adv_) between a liquid (water) and a solid is determined [56]. The system also allows the measurement of the contact angle of the fiber bundle, which has already been described in the publications of other authors [57,58]. During the measurements, the sample (in the form of fiber/rod) is stationary, and the vessel holder moves up (advancing cycle) and down (receding cycle). Each sample was repeatedly dipped in and withdrawn from the liquid vessel three times to measure a series of dynamic advancing contact angles. The dynamic contact angles at constant advancing velocities can be calculated from the Wilhelmy equation [59]:

(2) $$F=L\times \gamma \times {\cos\theta }_{\mathrm{Adv}}$$

where F is the force detected by the microbalance, L is the wetted length of the sample, θ_Adv_ is the dynamic contact angle. 

#### 2.2.7. In Vitro Study

##### Cell Culture and Experimental Groups

Human neuroblastoma SH-SY5Y cells (ATCC CRL-2266, Manassas, VA, USA) were cultured in high glucose DMEM (Life Technologies Ltd., Paisley, UK) supplemented with a 10% fetal bovine serum (FBS, Life Technologies Ltd., Paisley, UK) and 1% penicillin + streptomycin solution (Life Technologies Ltd., Paisley, UK). Cells were propagated in sterile cell culture flasks (75 cm^2^) and kept at 37 °C in a saturated humidity atmosphere containing 5% CO_2_. After reaching 80% confluency the cells were trypsinized (0.05% trypsin/EDTA, Life Technologies Ltd.)), counted (Bürker chamber) and seeded at a density of 8 × 10^4^ into 48-well plates containing the investigated materials (CF_FF, Pt wire, CF, and CF_PyC120) which were immobilized in the wells by quartz rings. Pt wire was used as a reference due to being the material from which conventional DBS electrodes are currently made whereas CF_FF, made of a core in the form of CF and a matrix in the form of phenol formaldehyde resin, was used as a positive control sample in the form of a rod of a size comparable to the investigated samples. Before cell seeding, the plates with materials and rings were sterilized with UV for 30 min. The control experimental group involved seeding wells containing only quartz rings. There were 3 replicates for each experimental group.

##### Cytotoxicity Assay

Twenty-four and forty-eight hours after cell seeding, 50 μL from each cell culture well was collected in a 96-well plate format to assess the potential cytotoxic effect of the studied materials in comparison to the control group. This parameter was measured by the Cytotoxicity Detection Kit (RocheDiagnostic, Mancheim, Germany) described previously [60]. The absorbance of each sample was measured after 15 min from reagent addition (25 μL/well) with a multi-well plate reader (Infinite^®^ M200 PRO, Tecan, Mannedorf, Switzerland) at 490 nm. After the subtraction of blank value (absorbance of cell culture medium) the data were normalized to the control group and are presented as the mean ± S.E.M. from three replicates. 

##### Live Cell Imaging

Twenty-four and forty-eight hours after cell seeding the plates were imaged using the differential interference contrast (DIC) light microscopy method. For this purpose, an inverted microscope AxioObserver (Carl Zeiss, Jena, Germany) was used, which was equipped with a white–black camera (Axio-CamMRm, Carl Zeiss, Jena, Germany). One microphotograph was taken for each well. 

##### Cell Viability Assay

Forty-eight hours after cell seeding, the WST-1 reagent was added to all experimental groups (in 2 replicates) as described previously [61]. After 60 min of incubation with the substrate, 100 μL of probe from each experimental group (in 2 replicates) was transferred to a 96-well plate. The absorbance of samples was measured with a multi-well plate reader (Infinite^®^ M200 PRO, Tecan) at 440 nm (measurement wavelength) and 630 nm (reference wavelength). Data (calculated difference between measurement and reference measurement) after subtraction for blank value (total damage, 1% Triton X-100 for 15 min) were normalized to the control group and are expressed as a percentage of the control ± S.E.M.

##### Scanning Electron Microscopy

At 48 h after cell seeding, the SH-SY5Y cells were fixed in 4% paraformaldehyde and then the samples were washed with PBS, treated with a 25% glutaraldehyde and 8% formaldehyde solution in a cacodylate buffer overnight, and then washed in cacodylate buffer. Next, the samples were dehydrated in increasing concentrations of ethanol (from 5% to 100%). All the dehydration steps were carried out at RT. The samples were finally dried using a CO_2_ critical point dryer, attached to the holders, and coated with a thin layer of carbon. Neuronal cells were analyzed using scanning electron microscopy (SEM) (NovaNanoSEM 200, FEI).

##### Statistical Analysis

Data were analyzed using Statistica software [62]. The analysis of variance (one- or two-way ANOVA) and post hoc Duncan test for multiple comparisons were used to show statistical significance with assumed *p* < 0.05.

## 3. Results and Discussion

### 3.1. Morphology and Microstructure of Rod-Shaped C/C Composite

**Figure 4:**
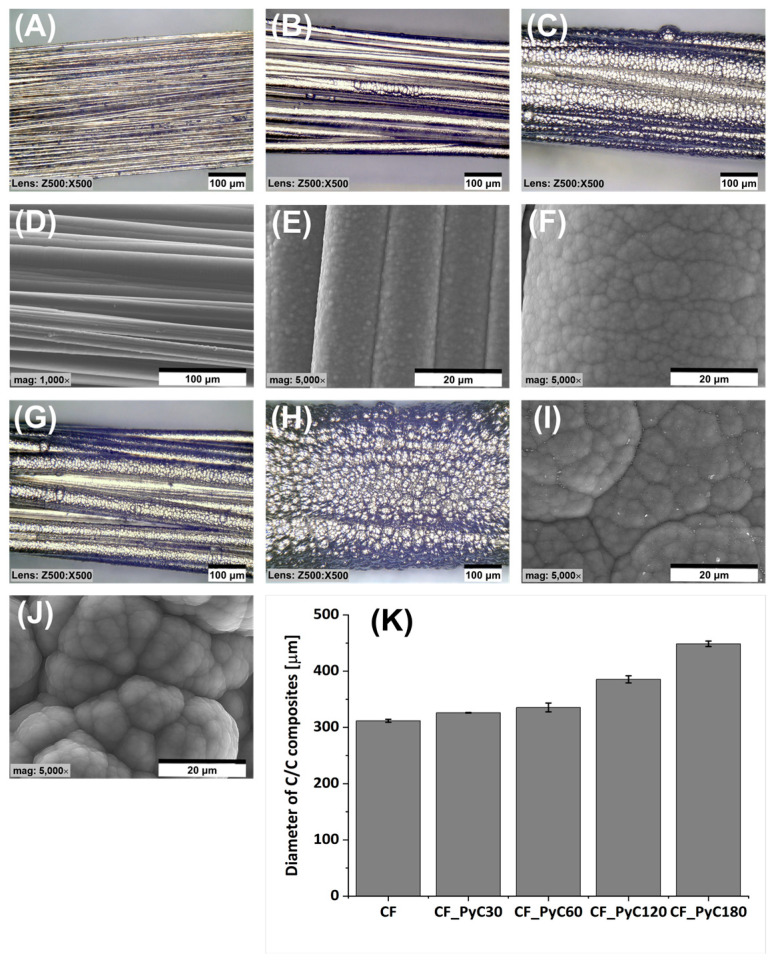
Morphology of a bundle of carbon fibers (**A**,**D**) and C/C composites after different durations of PyC synthesis (**B**,**E**) 30 s, (**C**,**F**) 60 s, (**G**,**I**) 120 s and (**H**,**J**) 180 s, diameter of C/C composites (**K**).

**Figure 5:**
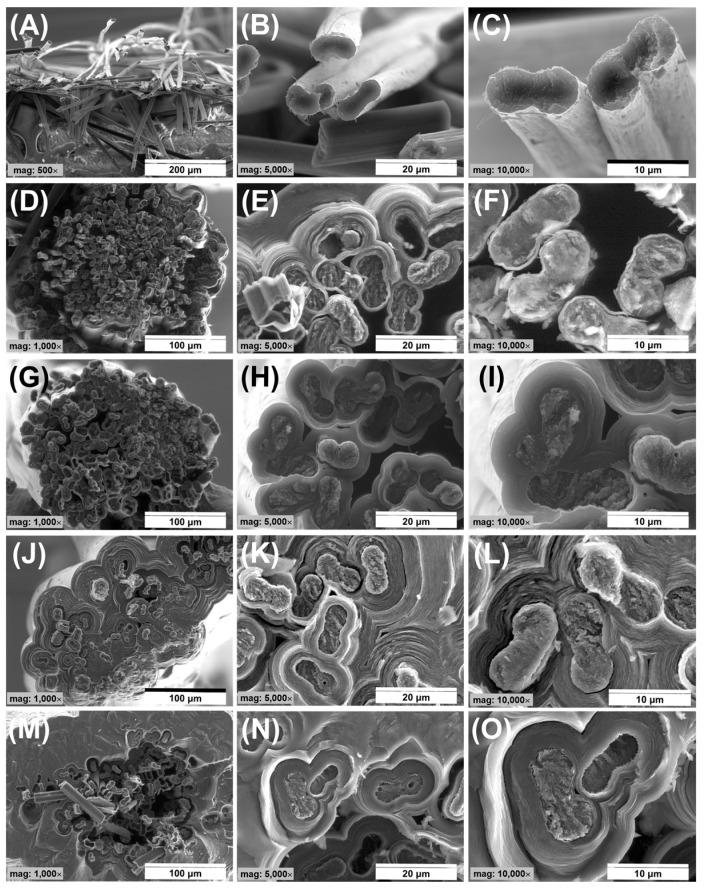
SEM analysis of cross-sections of the obtained C/C composites depending on the time of PyC synthesis, (**A**–**C**) bundle of CF, (**D**–**F**) CF_PyC30, (**G**–**I**) CF_PyC60, (**J**–**L**) CF_PyC120, (**M**–**O**) CF_PyC 180.

The morphology and microstructure of the obtained C/C composites were shown both in digital and SEM images (Figure 4 and Figure 5). The surface morphology of the C/C composites is rough, containing numerous ball-like protuberances characteristic of pyrocarbon [63]. The size of the spherical structures increases with the length of the synthesis time. The smallest of them are present in the case of PyC synthesis for 30 s (Figure 4B,E), and the largest at 180 s (Figure 4H,J). Also, with the increase in synthesis time, an increase in the PyC thickness on the surface is observed, which translates into an increase in the diameter of the C/C composites (Figure 4K). In addition, in the case of composites obtained in the time from 30 to 120 s, the morphology is preserved to some extent reflecting the fibrous form of the substrate itself. An increase in the synthesis time to 180 s causes the disappearance of this tendency, which is most likely related to a large increase in the thickness of the PyC. All composites were characterized by significant stiffness when compared to the bundle of carbon fibers (Figure 4A,D). This was observed especially at synthesis times of 60 s and more (Figure 4C,F–J).

The main factor that determined the choice of pyrocarbon synthesis conditions was to obtain C/C composites in which PyC would fill the spaces between individual fibers in the bundle, allowing on the one hand an increase in stiffness, while at the same time minimizing any increase in diameter of the material obtained in the form of rods. An important study allowing the observation of the degree of composite densification was the preparation of fractures and their examination using SEM (Figure 5). The analysis of the cross-sections of the obtained composites shows a significant difference between the samples, strongly correlated with the PyC synthesis time. Synthesis of PyC using the CVD method as a result of direct heating of a bundle of carbon fibers allowed us to observe that in the range of synthesis time from 30 s to 60 s a significant degree of porosity can be observed in the volume of a bundle of carbon fibers (Figure 5D–I). 

**Figure 6:**
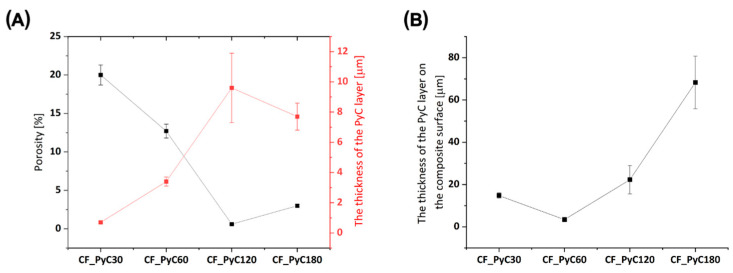
Porosity and thickness of the PyC around the fibers inside the bundle in a C/C composite (**A**); the thickness of the PyC layer on the composite surface (**B**).

**Figure 7:**
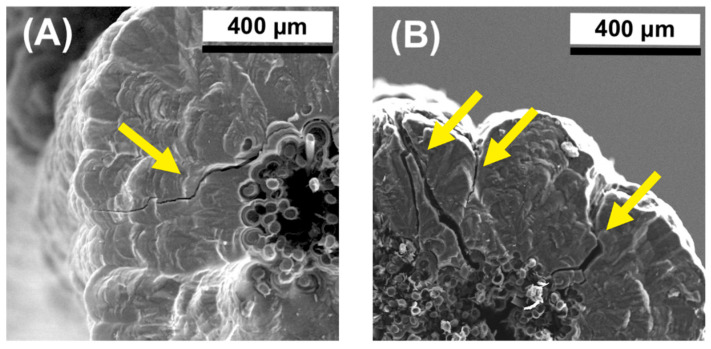
Crack propagation in the PyC layer in different places (**A**,**B**).

The pore size for these samples varies and is strongly dependent on the synthesis time. The highest porosity was observed for the shortest synthesis time, i.e., for 30 s, where the total porosity was about 20%. In turn, the lowest porosity was characteristic of the sample obtained at 120 s and was below 1% (Figure 6A). The decrease in porosity is closely related to the filling of the space between the individual fibers, which is also confirmed by the increase in the thickness of the PyC layers between the individual fibers in the volume of the bundle (Figure 6A). A short synthesis time means that the amount of synthesized PyC around the fibers is the smallest. It increases proportionally to the synthesis time and after 120 s, the spaces between the fibers are filled (Figure 5J–L), and the amount of pyrocarbon on the surface of the composites begins to increase (Figure 6B). An unquestionably unfavorable effect is observed after 180 s when the amount of PyC deposit significantly increases (Figure 5M and Figure 6B). Such an effect is undesirable due to a significant increase in the diameter of the composite, and the appearance of cracks in the layer (Figure 7 arrows), which may adversely affect the mechanical properties of the composite. In addition, such a large layer of pyrocarbon no longer fulfills the assumed requirements, i.e., creating a matrix in which carbon fibers are embedded. The pyrolytic carbon in these samples has a distinct laminar structure which promotes crack propagation extending along the laminar structure, parallel to the fiber surface. Crack propagation is even more likely as the thickness of the pyrocarbon layers increases. Crack propagation in laminar PyC has also been mentioned in other literature [46,64,65].

Analyzing the results of the assessment of the morphology and microstructure of the obtained C/C composites, the most favorable in terms of homogeneity of PyC distribution, is characterized by the CF_PyC120 sample. This composite was also characterized by the best handiness among the obtained samples in the form of a rod. It is this composite that will be subjected to further tests.

### 3.2. Structure of Rod-Shaped C/C Composite

**Figure 8:**
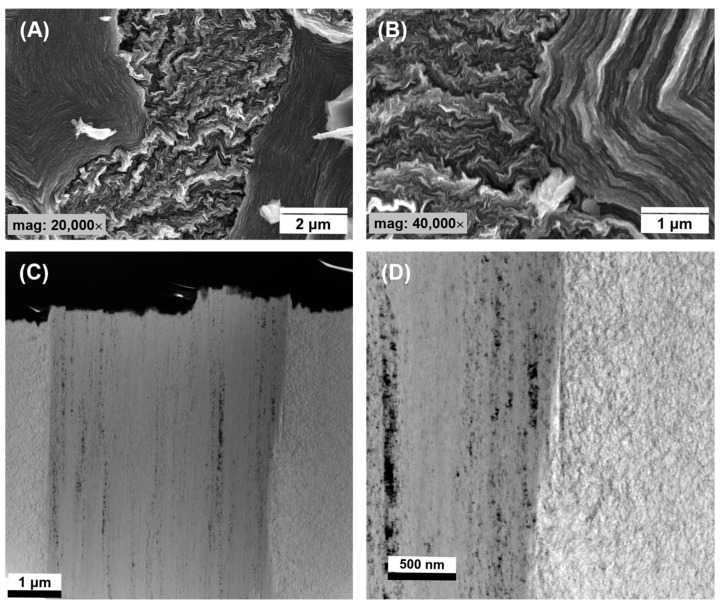
SEM (**A**,**B**) and STEM (**C**,**D**) morphologies of fracture surface of C/C composites.

**Figure 9:**
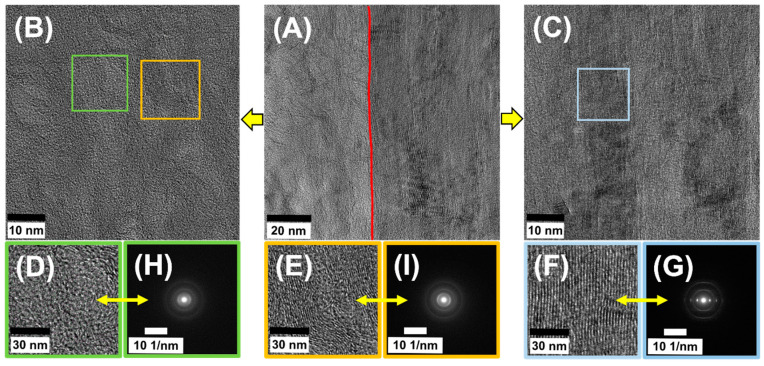
HRTEM images CF_PyC120 at the border between CF and PyC (**A**) composite, PyC in the CF_PyC120 composite (**B**,**D**,**E**), and CF in the CF_PyC120 composite (**C**,**F**) and SAEDs taken independently of HRTEM from two regions of PyC (**H**,**I**) and CF (**G**).

The structure and properties of the interface determine the adhesion between the fiber and the matrix. The mechanical properties of C/C composites are highly dependent on the load transfer at the fiber/matrix interface. A weak interface may impair the integrity of composites, whereas a strong bond may induce brittle fracture behavior [44,66]. For analyzing the microstructures close to the CF and PyC interface, SEM, TEM, and high-resolution TEM (HRTEM) were used. The analysis of the interface between the carbon fiber and the deposited PyC layer indicates good adhesion at the interface. The SEM microphotography indicates that the carbon fibers are surrounded by concentric pyrocarbon layers, and the boundary between these two phases is continuous (Figure 8A,B). It demonstrates that there is relatively good adhesion between the CF and the PyC. This is more visible from the scanning TEM HAADF-STEM micrographs shown in Figure 8C,D. As can be seen in the images the interface is continuous and free from any structural distortions. This is very evident from the area selected for illustration of the electron diffraction pattern (SAED) in the inset in Figure 9D. The corresponding orientation angles (OA), determined by the SAED pattern, indicate the existence of two different textures namely smooth laminar (SL) also called medium textured, OA = 76 ± 2° and dark laminar (DL) also called low textured, OA = 93 ± 2°. SL pyrocarbon is composed of wavy graphene layers, with strong distortions and curvatures. DL pyrolytic carbon is classified as isotropic carbon, although the preferred orientation of the pyrolytic carbon domains (texture) in this type of PyC is between typical isotropic (ISO) and that of typical low-textured ones, i.e., smooth laminar (SL) [43]. In the SEM images, we do not observe a clear difference in pyrocarbon morphology that would allow for a clear distinction between the SL and the DL pyrocarbons. SEM images indicate to a greater extent the presence of pyrocarbon SL (Figure 8A,B), which is characterized by a higher degree of texture parallel to the surface of the fiber [46,65]. 

Only the analysis of HRTEM images of the interface between the CF and PyC, as well as of the PyC itself, shows some differences in its structure and allows us to distinguish regions characteristic of SL and DL matrices (Figure 9). The analysis of HRTEM images of the PyC matrix clearly indicates the areas of occurrence of two types of pyrocarbon, i.e., with low and medium texturing (Figure 9B). In the HRTEM images, we can observe the interface between the fiber and the pyrocarbon. As in the case of TEM and SEM images, the interface between the phases is homogeneous, but there is a significant difference between the structure of the CF itself and the PyC (Figure 9A). The PyC matrix contains both low- and medium-textured domains, while the carbon fiber is characterized by high anisotropy and the clear arrangement of graphene layers parallel to the axis of the fiber (Figure 9C). The crystallographic structure of CF and PyC was assessed using the SAED technique (Figure 9) without the corresponding BF images; instead, the SAEDs are shown together with sample HRTEM micrographs to illustrate the structural features. The recorded diffraction patterns were characterized by the presence of three diffraction rings with scattering vectors of 3.0 nm^−1^, 4.8 nm^−1^, and 8.1 nm^−1^, assigned to the carbon planes (002), (100), and (110), respectively [67]. The diffraction rings in the case of CF showed a certain directional intensity distribution related to the (002) planes, indicating the anisotropic orientation of the crystal domains. In contrast, PyC showed a generally uniform intensity distribution with some slight direction indicating the isotropic orientation of the crystalline domains. The d_002_ value estimated from the diffraction patterns for CF was 3.39 Å, while for PyC it was 3.42 Å. The smaller value of the interplanar distances indicates a better ordering of the carbon structure in CF than in PyC, which is probably also related to the synthesis temperature of both types of carbon [68,69]. The carbon fibers were obtained at a temperature of 2000 °C, while the PyC synthesis temperature is between 1100–1200 °C. 

The high-resolution TEM HRTEM images, given for illustration in Figure 9, were taken with no objective aperture. In Figure 9A–C they are shown in a large field of view for a better illustration of the interface between the CF and PyC. The small squares marked in the images (Figure 9B) and (Figure 9C) indicate areas from which fast Fourier transforms (FFTs) were taken (Figure 9G,H). The FFTs are well in accordance with selected area electron diffraction patterns (SADPs), not shown, which were taken for statistics from different areas of the thin foil, from the respective regions corresponding to CF and PyC. Both the SADPs and FFTs indicate considerable changes in the structure between both phases. The results indicated more texture features and structural organization in the CF relative to PyC. The latter appeared more disarrayed and randomly organized. Regardless of the structural differences between both phases, however, the interface between the CF and PyC appeared largely coherent. It can be observed that some layers of graphene in the CF and smooth laminar pyrocarbon undulated together in the bonding area. This can improve the bond strength of the fiber with the pyrocarbon matrix acting like a hook. Therefore, the strength of the interfacial bonding of the fiber–PyC matrix can be strong [44]. 

When analyzing the possible mechanism of pyrocarbon growth in contact with a heated substrate, in this case, carbon fiber, the literature most often pays attention to such control parameters of the PyC deposition process as hydrocarbon concentration, residence time, surface area to volume of pore ratio (A/V) and temperature [70,71]. Initially, most of the work focused on the maturation of gases and the evolution of hydrocarbon pyrolysis decomposition products into small linear particles, which subsequently coalesced and combined into the synthesis of polycyclic aromatic hydrocarbons (PAHs) [70]. Over time, however, attention was paid to the role of the A/V ratio, and the concept of a nucleation and growth mechanism based on gas maturation and the concentration ratio between small linear molecules and polycyclic aromatic hydrocarbons (PAHs) was suggested [46,70,71,72]. The growth mechanism, usually obtained with short residence times and high A/V ratios, is based on the chemisorption of molecules (C_2_ or PAHs) in active sites at the edge of the graphene layer [73,74]. The second mechanism, i.e., nucleation, occurs most often in processes carried out with long residence times, high temperatures, and high concentrations of precursors and is associated with the physisorption of large PAH molecules on the surface of the substrate [72,74,75]. 

The PyC synthesis process takes place in the temperature range of 1100–1200 °C, however, it may be accompanied by various effects. In general, this temperature is relatively low, which is not sufficient to allow the maturation of the intermediate forms to produce large amounts of PAHs. Hence, the concentration of small linear/aromatic molecules increases, possibly reducing the formation of soot particles, and may also prevent the formation of five-member rings. This change in concentration will result in the deposition of PyC with a higher level of texture [70]. On the other hand, as was also described in one of the publications [70], if the PyC synthesis temperature is even lower, e.g., in the range of 1000–1100 °C, the amount of energy and intermediate compounds will not be enough to produce large aromatic molecules. This lack of large aromatic molecules will lead to the formation of five-member rings [72], thus producing PyC with lower levels of texture. At this stage, PyC formation will be completely controlled by the chemisorption of intermediate species at the graphene edges. Thus, in our particular case, we cannot clearly state which of the mechanisms is dominant, because, firstly, PyC deposition takes place in a temperature range that allows both nucleation and growth mechanisms to appear, and besides, temperature is not the only factor affecting this process. In addition to the aforementioned factors, the type of mechanism and microstructure may also depend on the construction of the reactor, and the distance between the nozzle and the substrate on which the deposition takes place, which has already been confirmed in the publications of other authors [70,71]. Therefore, in our case, the synthesis of PyC takes place through both mechanisms, as evidenced by the different microstructure of the obtained PyC.

**Figure 10:**
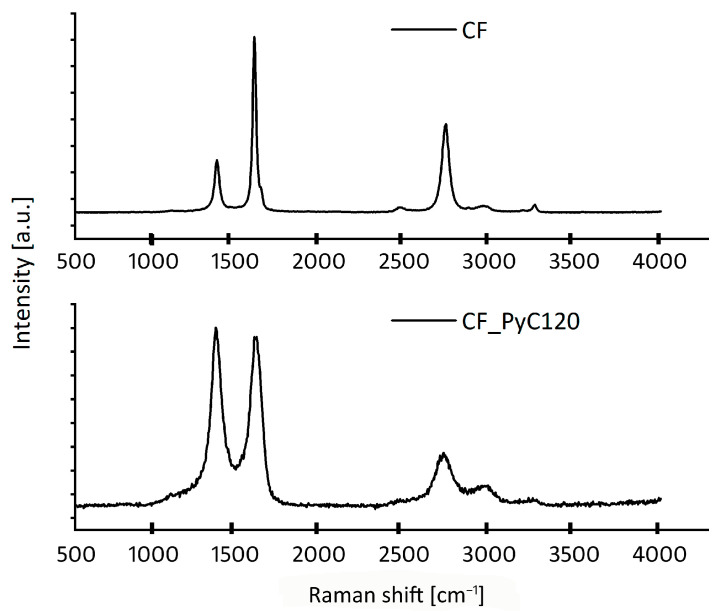
Raman spectra of carbon fibers (CF) and CF_PyC120 composite.

Raman spectroscopy was performed in order to evaluate the structure of the C/C composites and compare their properties to CF which is issued as the core of the composite. The Raman spectra of C/C composites (CF_PyC120) and carbon fibers are shown in Figure 10. 

Based on the Raman spectra, two characteristic D and G bands were observed for all samples. In the Raman spectra of carbon materials, two ranges of bands are observed, the first in the range of 1000–2000 cm^−1^ and the second in the range of 2000–3500 cm^−1^. In the former, there are two characteristic bands D and G. The D band occurs at the Raman shift of about 1350 cm^−1^ and is associated with the presence of defects that break the translational symmetry of the graphene sheet. The G band at about 1590 cm^−1^ is induced by the in-plane stretching vibrations of C=C bonds, which are attributed to longitudinal optical phonon mode at the center of the Brillouin zone of graphite. Near the G band, at Raman shift around 1620 cm^−1^, also the weak shoulder peak occurs, known as D’ band. This line originates from the double resonance intravalley scattering Raman processes activating phonons around Γ of the Brillouin zone of graphite, and it is one of the spectral feature characteristics of defective carbon materials. The separated D’ band is clearly visible only in the case of the CF sample, but it is present also in CF_PyC120, covered by the broadened G peak due to the lattice disorder. The exact contribution of this component was obtained using the Sadetzky five-band model described elsewhere [76]. In the second range of Raman spectra, there is a characteristic band at about 2650 cm^−1^, often also denoted as 2D, due to two phonons with opposite momentum in the highest optical branch near the K point of the Brillouin zone [77,78,79,80,81]. It is related to the stacking order of graphene sheets, sensitive to the electronic structure of carbon, and its intensity increases with the number of graphene layers [78,82]. In the second order region, weak overtone bands are also present for both samples—D+D’ (~2900 cm^−1^), D+D” (~2450 cm^−1^), and 2D’ (~3200 cm^−1^). Table 1 summarizes the structural parameters extracted from the Raman spectra of CF and CF_PyC120 samples. 

The results of Raman carbon fiber studies confirm the results obtained from high-resolution transmission microscopy (HRTEM) (Figure 9). Sharp and narrow characteristic bands testify to the highly crystalline character of the carbon fiber sample. The CF selected as a core of the C/C composite component is high-modulus, highly crystalline with high structural order. Such a structure of CF is evidenced by the greater intensity of the G band than the D band and the ratio of the integral intensities of the D and G bands (I_D_/I_G_) which is 0.36. This parameter is generally considered to be the basic indicator of the structural order of sp^2^ carbons, closely related to their electronic structure and crystallinity [80]. An additional parameter, often analyzed to determine the structure of the tested material, is the ratio of the integral intensities of the 2D and G bands (I_2D_/I_G_). A high value for this parameter indicates a three-dimensional long-range order and changes in the electronic structure associated with an increase in the concentration of charge carriers, as well as a large number of graphene layers in the tested material [81,82]. Structurally, C/C composites differ significantly from carbon fibers which are the core of these composites. PyC obtained by the CVD method at a temperature of 1100–1200 °C is characterized by a much lower structural order than carbon fibers, as evidenced by the I_D_/I_G_ and I_2D_/I_G_ parameters. The difference in the structure of the composite and carbon fibers is also evidenced by the size of the crystallite, namely the average lateral elongation of the graphene planes, which is more than four times greater in carbon fibers than in the C/C composite (Table 1).

**Figure 11:**
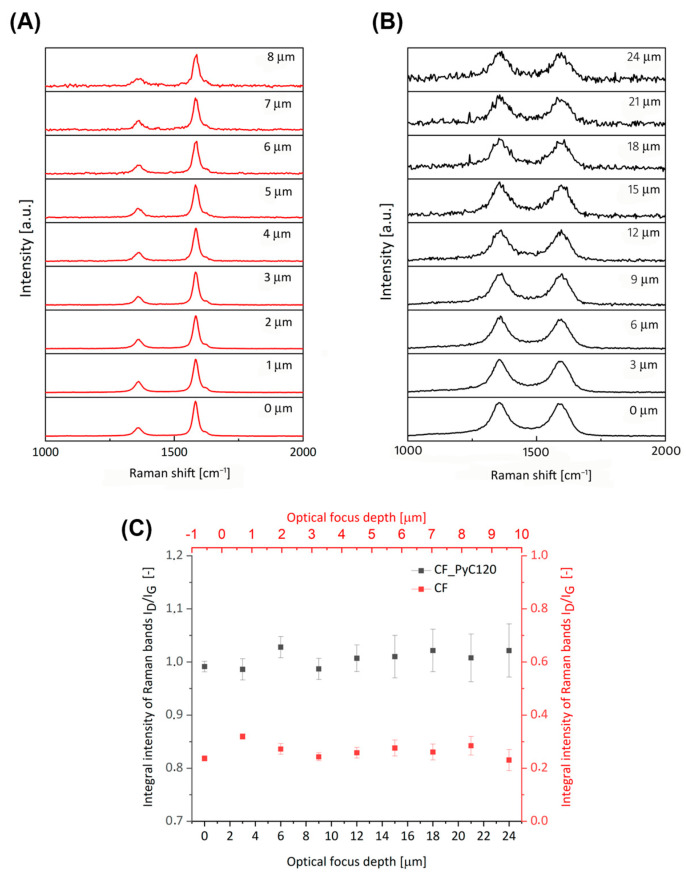
First-order Raman spectra of CF (**A**) and CF_PyC120 (**B**) composites collected at the various optical focus depths. (**C**) Depth profiles of the I_D_/I_G_ (areas) of CF and CF_PyC120 composite.

In order to answer the question of whether the conditions prevailing during the CVD synthesis affect the changes in the pyrolytic carbon structure, an analysis of the PyC at a depth was carried out. Optical focus depth profiling with a Raman confocal microscope was used to analyze the PyC from the outer surface to a depth of 24 µm (Figure 11B). The analyzed thickness in the case of PyC was dictated by its thickness on the surface of the fibers in the composite, which ranged between 20–30 µm. The same profiling was also carried out on carbon fiber, but to a depth of 8 µm, due to the diameter (longer diagonal) of the fiber (Figure 11A). For pyrolytic carbon, there is a slight decrease in signal intensity with increasing depth of focal, resulting mainly from the absorption of radiation depending on the absorption coefficient of the material, the depth of the optical focus, and the in-depth probe response parameter [77,83]. In order to quantify the structural changes at the depth of the PyC layer, the I_D_/I_G_ parameters were determined. Determination of these parameters did not cause problems, because no significant changes in peak broadening caused by noise were observed at the tested depth, therefore band areas were used to determine them (Figure 11B,C). There was a similar situation in the case of carbon fibers (Figure 11A,C).

The values of the I_D_/I_G_ parameters are at a similar level along with the depth of profiling of the tested samples. The obtained results confirm the structural homogeneity of the fibers themselves, which means that it is a commercial product. Whereas the results for the composite may confirm that the structural layer of PyC synthesized on the surface of carbon fibers is uniform in thickness throughout (Figure 11C). The obtained results are also confirmed by tests performed with the use of transmission electron microscopy (Figure 9).

### 3.3. Surface Chemistry of Rod-Shaped C/C Composite

**Figure 12:**
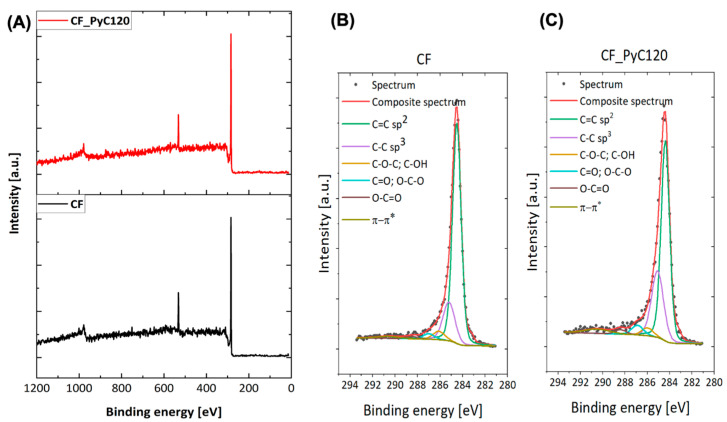
(**A**) XPS survey spectra of CF and CF_PyC120. Deconvoluted C1s peaks at (**B**) CF, (**C**) CF_PyC120.

The surface chemical composition of the carbon fibers (CF) and C/C composites (CF_PyC120) determined by the XPS is shown in Table 2. The main elements detected on the surfaces of these samples are carbon (C1s line) and oxygen (O1s line). The C1s core-level spectra for all samples were fitted with six components corresponding to C=C (sp^2^) type bonds (284.5 eV), C-C (sp^3^) bonds (285.3 eV), C-OH/C-O-C bonds (286.1 eV), C=O/O-C-O bonds (287.0 eV), O-C=O groups (288.5 eV) and π→π* satellite (291.0 eV) [12,31]. The latter is correlated with the graphitic character of the samples. The peak fitting of the C1s core level for CF and CF_PyC120 is presented in Figure 12.

The total carbon content in both analyzed samples is at a similar level. Nonetheless, analysis of the C1s peak indicates a higher content of carbon in hybridization sp^2^ in the CF as compared with CF_PyC120. The percentage of sp^2^ bonds is strictly related to the degree of graphitization of carbon and correlates well with the results obtained from HRTEM and Raman spectroscopy (Figure 9 and Figure 10). At the same time, for graphitized CF, the percentage of sp^3^ (C–C) bonds associated with the presence of defects in the carbon structure is lower than for the CF_PyC120 composite sample. Interestingly, in the case of carbon fibers, we observe a relatively high oxygen content, mainly in the form of C-O, C=O, and O-C-O bonds, higher than for the composite with a pyrolytic carbon matrix. On the one hand, this may indicate the presence of sizing in this type of fiber, but it may also be related to the presence of structural defects on the surface of the fiber, for example in the form of dangling bonds capable of reacting with atmospheric oxygen. The presence of defects on the fibers’ surface may also be evidenced by the presence of a rather large amount of bonds with sp^3^ hybridization (14.20%) for graphitized fibers.

**Figure 13:**
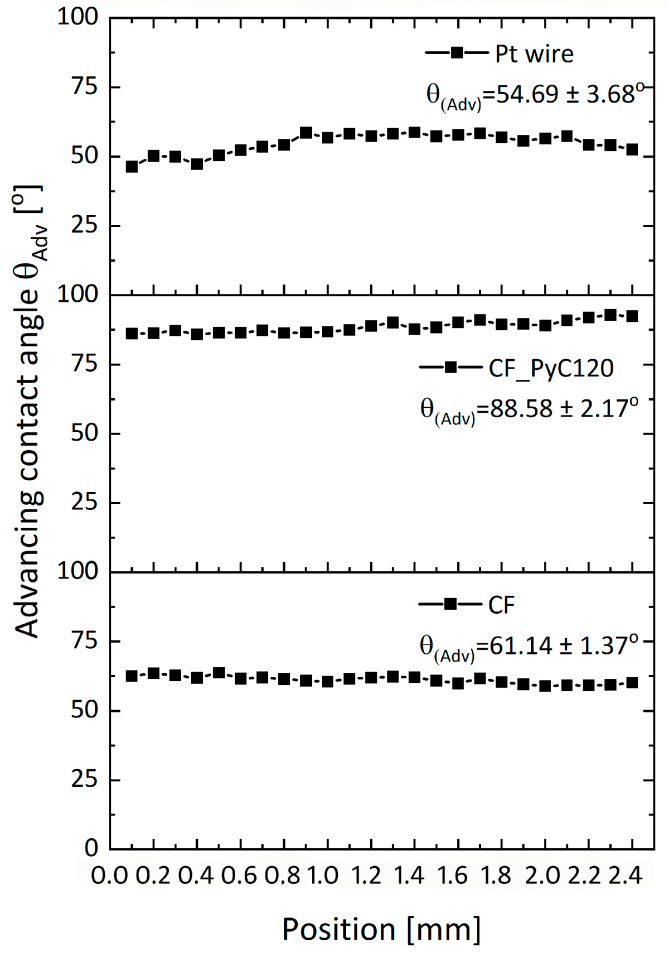
Typical measured θ_Adv_ versus position (mm) curves for CF tow, CF_PyC120, and Pt wire samples in water.

In order to assess the wettability of the surface of the tested materials, the Wilhelmy method was used, applying a tensiometer with a holder for testing single fibers with a diameter above 20 µm. This method is dedicated primarily to the analysis of solid samples, but it can also be used for bundles of fibers, as in the case of CF tow [57,58]. Wettability was evaluated for three types of samples, namely C/C composites, carbon fibers as the main reinforcing and directivity component of the composite, and a platinum (Pt) wire. The Pt wire was chosen because in the next study (in vitro study) it will be used as a reference due to the fact that it is currently the material from which electrodes for deep brain stimulation are most often made. The figures for individual values of the dynamic contact angle relative to the position during immersion of the samples in water and the results of the average values of the advancing angle θ_Adv_ are presented below (Figure 13).

The highest value of the contact angle is observed for the composite sample, which is 88.58 ± 2.17°. The values of the water contact angle for pyrolytic carbon, used primarily in medicine as a coating on the surface of bileaflet mechanical heart valve prostheses, are in the range of 86–110°. The exact value mainly depends on the synthesis temperature, types of precursor, and the presence of other elements, such as Si, used to improve mechanical parameters, mainly hardness and abrasion resistance [69,84,85]. In medicine, in particular for covering mechanical heart valves, low-temperature pyrolytic carbons are obtained at a temperature below 1500 °C [41]. Low-temperature isotropic carbon differs from isotropic carbon obtained at temperatures above 2000 °C not only in the degree of ordering of the carbon structure but also in the presence of heteroatoms which may affect, among other things, the degree of wettability of pyrolytic carbon. LTI carbon is characterized by a contact angle of about 90°; therefore, the value of the contact angle for PyC in CF_PyC120 composite confirms that we are dealing with low-temperature PyC. The wettability of the fiber bundle is higher than that of the composite sample, which is most likely due to the presence of sizing on the surface of carbon fibers. The presence of sizing on the surface of the fibers is also confirmed by the XPS test results. The value of the water contact angle for the tested fibers is about 61.14 ± 1.37°, which is consistent with the values presented in the literature for water contact angles of carbon fibers with sizing. The most frequently presented values of these angles in the literature are 62.5°, 65.8°, and about 71° depending on the research method used [57,58,86,87]. The first two values refer to the dynamic method, as in this paper. The lower contact angle of CF compared to the C/C composite may also be evidenced by the presence of oxygen groups associated with sizing as well as in combination with structural defects on the fiber surface, which is confirmed by the XPS test results (Table 2, Figure 12). A higher oxygen content in the CF sample is evidenced by, for example, a higher O/C ratio (Table 2).

The wettability of the Pt wire is 54.69 ± 3.68° and confirms data in the literature indicating the hydrophilic character of this material [88].

### 3.4. Biocompatibility of Rod-Shaped C/C Composite

The in vitro biological tests were aimed at the preliminary assessment of cytotoxicity and cell viability in contact with the manufactured materials. The SH-SY5Y cell line is derived from human neuroblastoma cells and is a widely accepted model of human neuronal-like cells [89,90]. This cell line, being of catecholaminergic phenotype, is often used as an in vitro model for neurotoxicity and neurodegenerative disorders. This line was selected for in vitro biological studies due to the intention to use the manufactured composites in the brain. The SH-SY5Y cell line is an excellent model for screening for the first assessment of the biocompatibility of the obtained C/C composites. To date, there have not been any studies in the literature regarding the preparation of such composites in the form of rods intended for the stimulation of nervous tissue cells, and equally so biological studies assessing their potential cytotoxicity. That is why these tests are so important, and the choice of the SH-SY5Y line in this case is certainly justified.

**Figure 14:**
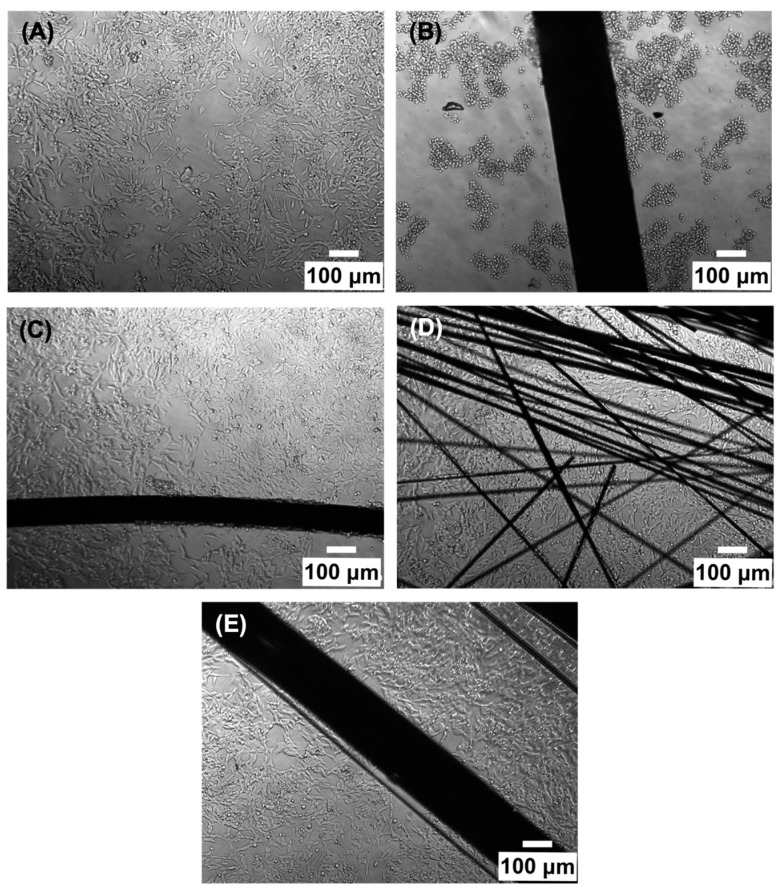
Differential interference contrast (DIC) images of SH-SY5Y cells in contact with (**A**) PS—negative control, (**B**) CF_FF—positive control, (**C**) Pt wire, (**D**) CF and (**E**) CF_PyC120 samples acquired 48 h after cell seeding.

**Figure 15:**
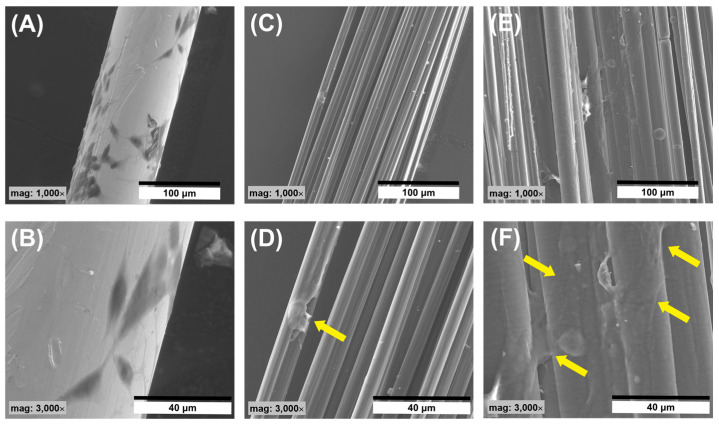
SEM micrographs of sample surfaces in contact with SH-SY5Y cells, (**A**,**B**) Pt wire, (**C**,**D**) CF, and (**E**,**F**) CF_PyC120.

**Figure 16:**
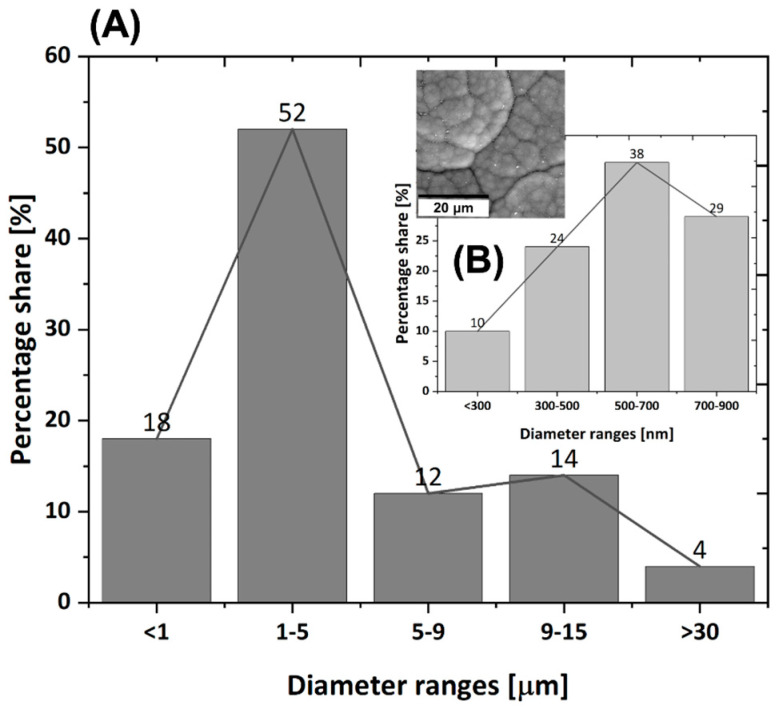
Size distribution of ball-like protuberances on the surface of CF-PyC120 composites; (**A**) size distribution of ball-like protuberances above 1 µm, (**B**) size distribution of ball-like protuberances above and below 1 µm. The numbers above the bars mean percentage share of ball-like protuberances on the surface of CF-PyC120 composites.

The in vitro tests were carried out for three types of samples, i.e., CF, CF_PyC120 composite, and Pt wire, which served as a reference sample. In addition, a positive control sample (CF_F-F composite) and a negative control sample (PS) were used. Both qualitative tests, i.e., mainly imaging in light (Figure 14) and scanning electron microscope (SEM) (Figure 15), were performed for the tested samples, as well as quantitative tests, i.e., viability using the WST-1 test and cytotoxicity using the lactate dehydrogenase (LDH) release test (Figure 16). 

**Figure 17:**
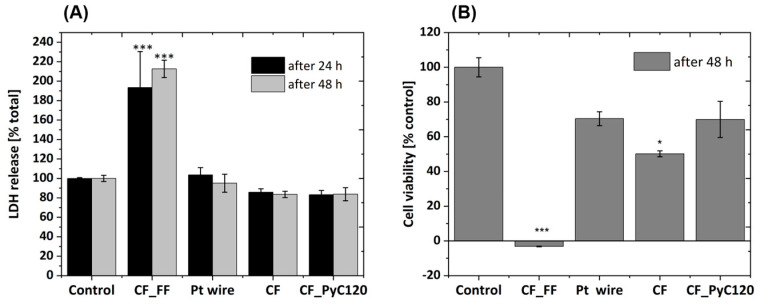
Quantitative biosafety assessment of the tested materials using cytotoxicity (**A**) and cell viability (**B**) biochemical assays. Cytotoxicity was assessed by LDH release assay and cell viability by WST-1 assay (detail in Material and Methods section). The data were normalized to the control group and are presented as a mean S.E.M. Two-way (**A**) and one-way (**B**) ANOVA. with post hoc Duncan test was used with * *p* < 0.05 and *** *p* < 0.001 vs. control group.

Based on the microscopic images shown in Figure 14, the morphology of SH-SY5Y cells in contact with samples can be assessed. Most of the cells in contact with a negative control sample (PS) have a polygonal, star shape; these cells are flattened, form clusters, and strongly adhere to the PS surface (Figure 14A). Cells in contact with the tested materials CF and CF_PyC120 after 48 h of culture also have a flattened, star shape, very similar in morphology to the cells on the PS control samples. Similar results can be observed for cells in contact with Pt wire. No morphological changes were observed in close or distant proximity to the sample compared to the negative control sample. In order to exclude the influence of the size and amount of the sample on the cellular response, a positive control sample (CF_FF) in the form of a rod of a size comparable to the analyzed samples was also prepared. Three repetitions were made for each test sample; in all three the amount of a given sample in the form of rods in each culture well was the same and amounted to three pieces. Significant differences in cell morphology and structure were observed for the positive control sample (CF_FF) compared to the negative control sample as well as to other samples. In this sample, the cells are showing signs of severe damage, evidenced by a smaller size, rounded shape, loose attachment to the surface, and agglomeration (Figure 14B). The negative impact of the positive sample is also evidenced by the results of cytotoxicity studies carried out using the LDH assay and cell viability in contact with samples (Figure 17). 

In addition, the analysis of SEM images allowed the observation of the behavior of cells in contact with the surface of the tested samples (Figure 15). Three types of samples were used for the tests, namely, a Pt wire as a reference, CFs, and a C/C composite. On the surface of the Pt wire, the presence of single flattened cells can be observed (Figure 15A,B). Due to the difference in the electron density of the tested materials, the SEM images of the cells on the surface of the Pt wire are by far the most visible. In the case of a sample of CFs, the number of cells adhering to the surface of the sample is much smaller compared to the Pt wire (Figure 15C,D). In this case, a single cell is seen, relatively flattened on the surface of the fiber bundle (Figure 15D, arrow). In the case of composite samples, it can be observed that the cells on the surface of the sample cover quite a large area and are well spread out (Figure 15E,F). The SEM images show the presence of single pseudopodia (Figure 15F arrows) of cells occupying more distant areas of the sample, which may indicate good SH-SY5Y cell adhesion to the sample surface. It can even be observed that the surface area occupied by the cells in contact with the CF_PyC120 sample is larger when compared to the reference sample (Pt wire). 

One probable reason for the better adhesion of cells to the surface of the composite sample is the larger diameter of this sample compared to carbon fibers, in which the diameter of a single fiber is between 4 µm and 12 µm (depending on the direction) (Figure 1). On such a surface, the cell has more opportunities for proper anchoring and better adhesion than on the surface of a sample with a small diameter and which is also elongated. When analyzing the influence of the surface properties of the tested samples on the adhesion of cells, the first parameter that appears is wettability. The highest wettability can be observed for carbon fibers, although the remaining samples also have a hydrophilic surface (Figure 13). The surface of the C/C composite sample is the least wettable but having analyzed the behavior of cells on this surface, it can be concluded that this is not the most important parameter determining the cellular response. The surface of the CF_PyC120 sample also contains some ball-like protuberances, cauliflower-like structures of different sizes (Figure 16), which may contribute to better adhesion by creating additional sites for cell attachment to the sample surface. In the tested C/C composite sample, we can talk about hierarchical roughness containing both precipitates with dimensions of several to several dozen micrometers (form 1 µm to >30 µm, Figure 16A), as well as roughness in the nanometric scale (in the range from <300 nm to 900 nm, Figure 16B). Analyzing the data from the literature, the presence of roughness in the micro- and nanoscale seems to be the most desirable property with regard to cellular response and the effect on cell adhesion [91,92,93].

The cytotoxicity of the tested materials was determined on the basis of the LDH release test. Two-way ANOVA statistical analysis of LDH test results demonstrated the effects of the types of materials investigated, but not the time of incubation with the tested materials. We demonstrated a significant cytotoxic effect of the (CF_FF) sample at both of the studied time points (24 and 48 h), whereas the other materials were non-cytotoxic to SH-SY5Y cells when compared to the control group (Figure 17A).

The one-way ANOVA statistical analysis of cell viability data confirmed the severe cell-damaging effect of CF_FF found in the LDH test and light microscopy. Moreover, a significant reduction (about 50%) was observed for the CF samples and some tendency towards the reduction of cell viability (by about 30%) was also noted for the Pt wire and CF_PyC120 samples (Figure 17B). This was probably induced by the presence of material in the wells which blocked cell proliferation when compared to the control group. On this basis, it can be concluded that the PyC obtained by the CVD method in the resistance heating system improves cell viability compared to high-modulus CF without any modification. In turn, the high level of cytotoxicity for the CF_FF control sample also results in a significant decrease in cell viability, which confirms the negative effect of this sample on cellular response. Analyzing the effect of the individual components of the C/C composite on the cellular response, it can be concluded that none of them, i.e., neither carbon fiber nor pyrolytic carbon, have a negative impact on the cellular response of SH-SY5Y cells. Carbon fibers have been of interest in various areas of medicine for many years; these are primarily applications in the area of bone, cartilage, ligament, and tendon reconstructions [83]. In this area, the use of fibrous forms alone met with great enthusiasm at first, but over time it turned out that the biocompatibility of these materials is limited, this results primarily from the types of fiber used. Therefore, further applications of carbon fibers focused on their use as reinforcements in polymer composites. Lower cell viability in contact with CF compared to Pt and C/C composites may be, as mentioned earlier, the result of poorer cell adhesion to their surface, which was observed by analyzing the SEM results. Another factor that may also affect cell viability is the structure of the tested CFs. In the literature on the biocompatibility of CFs, attention is paid to the type of carbon fiber, whether they are high-modulus fibers, i.e., high-crystalline, or low-modulus, i.e., low-crystalline. Generally, the more crystalline the samples of carbon fibers and the more ordered their structure, the worse the biological response [94]. The authors of these papers indicated that carbon fibers with higher crystallinity and a better-organized graphite structure were assimilated by the body with more difficulty, and small particles coming from these materials were found in the regional lymph nodes. The carbon fibers used in this work are high-modulus fibers with a high degree of crystallinity, which was confirmed by HRTEM tests and Raman spectroscopy (Figure 9 and Figure 10). These fibers are also dominated by carbon with sp^2^ hybridization, which proves the ordered structure of this material. Pyrolytic carbon constituting the matrix of the C/C composite, in turn, is characterized by a more amorphous structure and a higher content of carbon with sp^3^ hybridization than in the case of carbon fibers (Table 2). This amorphous structure is associated with the presence of structural defects, capable of interacting with the surrounding environment, including protein in the culture medium or on cellular membranes [95,96,97]. Therefore, these factors may also affect cell viability and demonstrate higher biocompatibility of C/C composites with PyC matrix. While carbon fibers in applications for the stimulation of nervous tissue as microelectrodes have been the subject of research [25,26], so far pyrolytic carbon has not been tested in relation to nervous tissue cells, so these results can be considered pioneering.

## 4. Conclusions

This study is focused on creating carbon–carbon composites using carbon fibers and pyrolytic carbon in the form of rods. These composites are being investigated as potential materials for use with nerve tissue cells. Since these materials have not been previously considered for treating neurodegenerative diseases, each step of their preparation is crucial. The first objective was to develop a method for obtaining rod-shaped C/C composites with dimensions below 1 mm. To achieve this, a non-standard approach using direct electrical heating of a bundle of carbon fibers in the CVD method was employed. The study examined the influence of different synthesis times (30 s, 60 s, 120 s, and 180 s) on the quality of the resulting composites. The surface of the cross-sections of the samples was analyzed through SEM to assess sample compaction, porosity, PyC layer thickness, and composite rod diameters. Based on these analyses, the most suitable conditions for the synthesis of C/C composites were identified, specifically a sample designated as CF_PyC_120, synthesized for 120 s using methane as the carbonaceous gas.

Another essential aspect of the research was the evaluation of the structure of the obtained composite rods, particularly the PyC matrix. High-resolution transmission electron microscopy (HRTEM), selected area electron diffraction (SAED), and Raman spectroscopy were employed to determine the degree of crystallinity in the pyrocarbon structure, measure interplanar distance (d_002_), and establish the size of crystallites in both the PyC phase and fibers. The orientation angle (OA), which indicates the texture of PyC, was also analyzed, revealing the presence of two distinct textures: smooth laminar and dark laminar. The interface structure influences the adhesion between the fibers and matrix, which can significantly impact the mechanical properties of C/C composites and load transfer at the fiber/matrix interface. Understanding the structural parameters also affects the electrical properties of the composites, which will be studied in subsequent research stages.

In addition to structural parameters, the study investigated the morphology and microstructure of the surface of the C/C composites in the form of rods, as well as their surface chemistry, which affects factors like sample wettability and cellular response in vitro. The X-ray photoelectron spectroscopy (XPS) method and tensiometer were used to determine these parameters. Furthermore, since these materials had not been tested before for their response to nerve tissue cells, it was crucial to assess their toxicity and viability against neural cells. The SH-SY5Y cell line, commonly used in neurological experiments, was used as a model for this purpose. Cytotoxicity was examined using the LDH release test, while viability studies were conducted using the WST-1 test. The results showed that both carbon fibers and pyrolytic carbon in the C/C composites did not negatively impact the cellular response of SH-SY5Y cells. Cell viability on the surface of the composite was at a similar level to that of the reference sample, which was a Pt wire. In turn, assessing the morphology of cells in contact with the tested CF_PyC_120 composite using SEM and DIC light microscopy methods, it can be concluded that after culture they have a flattened and star-like shape, morphologically very similar to the cells on the PS control samples. Also, no morphological changes were observed in close or distant proximity to the sample when compared to the negative control sample. Interestingly, in the case of the C/C composite samples, it can also be observed that the cells are well distributed on their surface, with visible single pseudopodia of cells occupying more distant areas of the sample, which may indicate good adhesion of SH-SY5Y cells to the sample surface. It can even be observed that the surface occupied by cells in contact with the CF_PyC120 sample is larger than on the reference sample (Pt wire). Good adhesion of cells to the surface of the CF_PyC_120 composite may be determined by the hierarchical roughness of the composite surface as well as its amorphous character, manifested by the presence of structural defects, such as dangling bonds capable of interacting with the surrounding biological environment.

## Figures and Tables

**Table 1:** Structural parameters obtained from Raman spectra of CF and CF_PyC120.

Sample	I_D_	I_G_	I_2D_	I_D_/I_G_	I_2D_/I_G_	L_a_ [nm]
**CF**	6148	17,107	15,937	0.36	0.93	37.88
**CF_PyC120**	7102	6844	3739	1.04	0.55	13.12

The values of L_a_ were obtained from Cancado Equation (1).

**Table 2:** Surface chemistry of CF and CF_PyC120 obtained from XPS analysis.

Sample	Elemental Composition (%)		(-)	Deconvolution of the C1s Spectra (%)					
	C	O	O/C	284.5 eV C=C (sp^2^)	285.3 eV C-C (sp^3^)	286.1 eV C-O, C-OH	287.0 eV C=O, O-C-O	288.5 eV O-C=O	291.0 eV π→π*
**CF**	87.60	12.40	0.14	65.90	14.20	3.10	2.00	0.90	1.50
**CF_PyC120**	90.30	9.70	0.11	56.10	23.10	2.80	3.40	1.60	3.30

## Data Availability

The data presented in this study are available from the corresponding author upon request.

## References

[1] Brown R.C., Lockwood A.H., Sonawane B.R. (2005). Neurodegenerative Diseases: An Overview of Environmental Risk Factors. Environ. Health Perspect..

[2] Checkoway H., Lundin J.I., Kelada S.N. (2011). Neurodegenerative diseases. IARC Sci. Publ..

[3] Chi H., Chang H.-Y., Sang T.-K. (2018). Neuronal Cell Death Mechanisms in Major Neurodegenerative Diseases. Int. J. Mol. Sci..

[4] Poddar K.M., Chakraborty A., Banerjee S. (2021). Neurodegeneration: Diagnosis, Prevention, and Therapy. Oxidoreductase.

[5] Hariz M., Blomstedt P. (2022). Deep brain stimulation for Parkinson’s disease. J. Intern. Med..

[6] Kim E., Kim S., Kwon Y.W., Seo H., Kim M., Chung W.G., Park W., Song H., Lee D.H., Lee J. (2023). Electrical stimulation for therapeutic approach. Interdiscip. Med..

[7] Malek N. (2019). Deep Brain Stimulation in Parkinson’s Disease. Neurol. India.

[8] Kolaya E., Firestein B.L. (2021). Deep brain stimulation: Challenges at the tissue-electrode interface and current solutions. Biotechnol. Prog..

[9] Arcot Desai S., Gutekunst C.-A., Potter S.M., Gross R.E. (2014). Deep brain stimulation macroelectrodes compared to multiple microelectrodes in rat hippocampus. Front. Neuroeng..

[10] Hickey P., Stacy M. (2016). Deep Brain Stimulation: A Paradigm Shifting Approach to Treat Parkinson’s Disease. Front. Neurosci..

[11] Polikov V.S., Block M.L., Fellous J.-M., Hong J.-S., Reichert W.M. (2006). In vitro model of glial scarring around neuroelectrodes chronically implanted in the CNS. Biomaterials.

[12] McConnell G.C., Rees H.D., Levey A.I., Gutekunst C.-A., Gross R.E., Bellamkonda R.V. (2009). Implanted neural electrodes cause chronic, local inflammation that is correlated with local neurodegeneration. J. Neural Eng..

[13] Wellman S.M., Li L., Yaxiaer Y., McNamara I., Kozai T.D.Y. (2019). Revealing Spatial and Temporal Patterns of Cell Death, Glial Proliferation, and Blood-Brain Barrier Dysfunction Around Implanted Intracortical Neural Interfaces. Front. Neurosci..

[14] Usoro J.O., Sturgill B.S., Musselman K.C., Capadona J.R., Pancrazio J.J. (2021). Intracortical Microelectrode Array Unit Yield under Chronic Conditions: A Comparative Evaluation. Micromachines.

[15] Cherry J.D., Olschowka J.A., O’Banion M.K. (2014). Neuroinflammation and M2 microglia: The good, the bad, and the inflamed. J. Neuroinflamm..

[16] Karumbaiah L., Saxena T., Carlson D., Patil K., Patkar R., Gaupp E.A., Betancur M., Stanley G.B., Carin L., Bellamkonda R.V. (2013). Relationship between intracortical electrode design and chronic recording function. Biomaterials.

[17] Mohammed M., Ivica N., Bjartmarz H., Thorbergsson P.T., Pettersson L.M.E., Thelin J., Schouenborg J. (2022). Microelectrode clusters enable therapeutic deep brain stimulation without noticeable side-effects in a rodent model of Parkinson’s disease. J. Neurosci. Methods.

[18] Tian G., Yang D., Chen C., Duan X., Kim D.-H., Chen H. (2023). Simultaneous Presentation of Dexamethasone and Nerve Growth Factor via Layered Carbon Nanotubes and Polypyrrole to Interface Neural Cells. ACS Biomater. Sci. Eng..

[19] Rodrigues A.F., Tavares A.P.M., Simões S., Silva R.P.F.F., Sobrino T., Figueiredo B.R., Sales G., Ferreira L. (2023). Engineering graphene-based electrodes for optical neural stimulation. Nanoscale.

[20] Lim J., Lee S., Kim J., Hong J., Lim S., Kim K., Kim J., Yang S., Yang S., Ahn J.-H. (2023). Hybrid graphene electrode for the diagnosis and treatment of epilepsy in free-moving animal models. NPG Asia Mater..

[21] Nekounam H., Samadian H., Golmohammadi H., Asghari F., Shokrgozar M.A., Ahadian S., Majidi R.F. (2023). Carbon nanofibers fabrication, surface modifications, and application as the innovative substrate for electrical stimulation of neural cell differentiation. Surf. Interfaces.

[22] Hejazi M.A., Tong W., Stacey A., Soto-Breceda A., Ibbotson M.R., Yunzab M., Maturana M.I., Almasi A., Jung Y.J., Sun S. (2020). Hybrid diamond/ carbon fiber microelectrodes enable multimodal electrical/chemical neural interfacing. Biomaterials.

[23] Dresvyanina E.N., Tagandurdyyeva N.A., Kodolova-Chukhontseva V.V., Dobrovol’skaya I.P., Kamalov A.M., Nashchekina Y.A., Nashchekin A.V., Ivanov A.G., Yukina G.Y., Yudin V.E. (2023). Structure and Properties of Composite Fibers Based on Chitosan and Single-Walled Carbon Nanotubes for Peripheral Nerve Regeneration. Polymers.

[24] Pi W., Zhang Y., Li L., Li C., Zhang M., Zhang W., Cai Q., Zhang P. (2022). Polydopamine-coated polycaprolactone/carbon nanotube fibrous scaffolds loaded with brain-derived neurotrophic factor for peripheral nerve regeneration. Biofabrication.

[25] Hejazi M., Tong W., Ibbotson M.R., Prawer S., Garrett D.J. (2021). Advances in Carbon-Based Microfiber Electrodes for Neural Interfacing. Front. Neurosci..

[26] Devi M., Vomero M., Fuhrer E., Castagnola E., Gueli C., Nimbalkar S., Hirabayashi M., Kassegne S., Stieglitz T., Sharma S. (2021). Carbon-based neural electrodes: Promises and challenges. J. Neural Eng..

[27] Bhatt P., Goe A. (2017). Carbon Fibres: Production, Properties and Potential Use. Mater. Sci. Res. India.

[28] Gillis W.F., Lissandrello C.A., Shen J., Pearre B.W., Mertiri A., Deku F., Cogan S., Holinski B.J., Chew D.J., White A.E. (2018). Carbon fiber on polyimide ultra-microelectrodes. J. Neural Eng..

[29] Manciu F.S., Oh Y., Barath A., Rusheen A.E., Kouzani A.Z., Hodges D., Guerrero J., Tomshine J., Lee K.H., Bennet K.E. (2019). Analysis of Carbon-Based Microelectrodes for Neurochemical Sensing. Materials.

[30] Lee Y., Kong C., Chang J.W., Jun S.B. (2019). Carbon-Fiber Based Microelectrode Array Embedded with a Biodegradable Silk Support for In Vivo Neural Recording. J. Korean Med. Sci..

[31] Dunn J.F., Tuor U.I., Kmech J., Young N.A., Henderson A.K., Jackson J.C., Valentine P.A., Teskey G.C. (2009). Functional brain mapping at 9.4T using a new MRI-compatible electrode chronically implanted in rats. Magn. Reson. Med..

[32] Cruttenden C.E., Taylor J.M., Hu S., Zhang Y., Zhu X.-H., Chen W., Rajamani R. (2017). Carbon nano-structured neural probes show promise for magnetic resonance imaging applications. Biomed. Phys. Eng. Express.

[33] Huffman M.L., Venton B.J. (2009). Carbon-fiber microelectrodes for in vivo applications. Analyst.

[34] Letner J.G., Patel P.R., Hsieh J.-C., Smith Flores I.M., della Valle E., Walker L.A., Weiland J.D., Chestek C.A., Cai D. (2023). Post-explant profiling of subcellular-scale carbon fiber intracortical electrodes and surrounding neurons enables modeling of recorded electrophysiology. J. Neural Eng..

[35] Zhao S., Li G., Tong C., Chen W., Wang P., Dai J., Fu X., Xu Z., Liu X., Lu L. (2020). Full activation pattern mapping by simultaneous deep brain stimulation and fMRI with graphene fiber electrodes. Nat. Commun..

[36] Bennet K.E., Tomshine J.R., Min H.-K., Manciu F.S., Marsh M.P., Paek S.B., Settell M.L., Nicolai E.N., Blaha C.D., Kouzani A.Z. (2016). A Diamond-Based Electrode for Detection of Neurochemicals in the Human Brain. Front. Hum. Neurosci..

[37] More R.B., Haubold A.D., Bokros J.C. (2013). Pyrolytic Carbon for Long-Term Medical Implants. Biomaterials Science.

[38] Li A., Norinaga K., Zhang W., Deutschmann O. (2008). Modeling and simulation of materials synthesis: Chemical vapor deposition and infiltration of pyrolytic carbon. Compos. Sci. Technol..

[39] Forti S., Lunelli L., Della Volpe C., Siboni S., Pasquardini L., Lui A., Canteri R., Vanzetti L., Potrich C., Vinante M. (2011). Hemocompatibility of pyrolytic carbon in comparison with other biomaterials. Diam. Relat. Mater..

[40] Daecke W., Veyel K., Wieloch P., Jung M., Lorenz H., Martini A.-K. (2006). Osseointegration and Mechanical Stability of Pyrocarbon and Titanium Hand Implants in a Load-Bearing In Vivo Model for Small Joint Arthroplasty. J. Hand Surg. Am..

[41] Stanley J., Klawitter J., More R. (2008). Replacing joints with pyrolytic carbon. Joint Replacement Technology.

[42] Norinaga K., Deutschmann O., Saegusa N., Hayashi J. (2009). Analysis of pyrolysis products from light hydrocarbons and kinetic modeling for growth of polycyclic aromatic hydrocarbons with detailed chemistry. J. Anal. Appl. Pyrolysis.

[43] Drescher M., Hüttinger K.J., Dormann E. (2003). Pyrolytic carbon layers—An electron spin resonance analysis. Carbon.

[44] He Y.-G., Li K.-Z., Li H.-J., Wei J.-F., Fu Q.-G., Zhang D.-S. (2010). Effect of interface structures on the fracture behavior of two-dimensional carbon/carbon composites by isothermal chemical vapor infiltration. J. Mater. Sci..

[45] Oku T. (2003). Carbon/Carbon Composites and Their Properties. Carbon Alloys.

[46] Reznik B., Gerthsen D., Hüttinger K.J. (2001). Micro- and nanostructure of the carbon matrix of infiltrated carbon fiber felts. Carbon.

[47] Xu X., Ouyang T., Zeng L., Chai L. (2018). Study on the Pyrolytic Carbon Generated by the Electric Heating CVD Method. J. Wuhan Univ. Technol. Sci. Ed..

[48] Kovalevich J., Langford D. (2013). Considerations for the Use of SH-SY5Y Neuroblastoma Cells in Neurobiology. Methods Mol. Biol..

[49] Jantas D., Chwastek J., Malarz J., Stojakowska A., Lasoń W. (2020). Neuroprotective Effects of Methyl Caffeate against Hydrogen Peroxide-Induced Cell Damage: Involvement of Caspase 3 and Cathepsin D Inhibition. Biomolecules.

[50] Ruffels J., Griffin M., Dickenson J.M. (2004). Activation of ERK1/2, JNK and PKB by hydrogen peroxide in human SH-SY5Y neuroblastoma cells: Role of ERK1/2 in H_2_O_2_-induced cell death. Eur. J. Pharmacol..

[51] Jantas D., Piotrowski M., Lason W. (2015). An Involvement of PI3-K/Akt Activation and Inhibition of AIF Translocation in Neuroprotective Effects of Undecylenic Acid (UDA) Against Pro-Apoptotic Factors-Induced Cell Death in Human Neuroblastoma SH-SY5Y Cells. J. Cell. Biochem..

[52] Reznik B., Hüttinger K. (2002). On the terminology for pyrolytic carbon. Carbon.

[53] Meadows P.J., López-Honorato E., Xiao P. (2009). Fluidized bed chemical vapor deposition of pyrolytic carbon—II. Effect of deposition conditions on anisotropy. Carbon.

[54] Meier R.J. (2005). On art and science in curve-fitting vibrational spectra. Vib. Spectrosc..

[55] Cançado L.G., Takai K., Enoki T., Endo M., Kim Y.A., Mizusaki H., Jorio A., Coelho L.N., Magalhães-Paniago R., Pimenta M.A. (2006). General equation for the determination of the crystallite size La of nanographite by Raman spectroscopy. Appl. Phys. Lett..

[56] Tiab D., Donaldson E.C. (2012). Wettability. Petrophysics.

[57] Qiu S., Fuentes C.A., Zhang D., Van Vuure A.W., Seveno D. (2016). Wettability of a Single Carbon Fiber. Langmuir.

[58] Wang J., Fuentes C.A., Zhang D., Wang X., Van Vuure A.W., Seveno D. (2017). Wettability of carbon fibres at micro- and mesoscales. Carbon.

[59] Yuan Y., Lee T.R. (2013). Contact Angle and Wetting Properties. Surface Science Techniques.

[60] Fraczek-Szczypta A., Jantas D., Ciepiela F., Grzonka J. (2020). Graphene oxide-conductive polymer nanocomposite coatings obtained by the EPD method as substrates for neurite outgrowth. Diam. Relat. Mater..

[61] Jantas D., Malarz J., Le T.N., Stojakowska A. (2021). Neuroprotective Properties of Kempferol Derivatives from Maesa membranacea against Oxidative Stress-Induced Cell Damage: An Association with Cathepsin D Inhibition and PI3K/Akt Activation. Int. J. Mol. Sci..

[62] StatSoft (2017). Statistica.

[63] Reznik B., Norinaga K., Gerthsen D., Deutschmann O. (2006). The effect of cooling rate on hydrogen release from a pyrolytic carbon coating and its resulting morphology. Carbon.

[64] Ren J., Li K., Zhang S., Yao X., Tian S. (2015). Preparation of carbon/carbon composite by pyrolysis of ethanol and methane. Mater. Des..

[65] López-Honorato E., Meadows P.J., Xiao P., Marsh G., Abram T.J. (2008). Structure and mechanical properties of pyrolytic carbon produced by fluidized bed chemical vapor deposition. Nucl. Eng. Des..

[66] Tezcan J., Ozcan S., Gurung B., Filip P. (2008). Measurement and analytical validation of interfacial bond strength of PAN-fiber-reinforced carbon matrix composites. J. Mater. Sci..

[67] Schierholz R., Kröger D., Weinrich H., Gehring M., Tempel H., Kungl H., Mayer J., Eichel R.-A. (2019). The carbonization of polyacrylonitrile-derived electrospun carbon nanofibers studied by in situ transmission electron microscopy. RSC Adv..

[68] Zambrzycki M., Piech R., Raga S.R., Lira-Cantu M., Fraczek-Szczypta A. (2023). Hierarchical carbon nanofibers/carbon nanotubes/NiCo nanocomposites as novel highly effective counter electrode for dye-sensitized solar cells: A structure-electrocatalytic activity relationship study. Carbon.

[69] Boehm R.D., Jin C., Narayan R.J. (2017). Carbon and Diamond. Comprehensive Biomaterials II.

[70] López-Honorato E., Meadows P.J., Xiao P. (2009). Fluidized bed chemical vapor deposition of pyrolytic carbon—I. Effect of deposition conditions on microstructure. Carbon.

[71] Hu Z.J., Zhang W.G., Hüttinger K.J., Reznik B., Gerthsen D. (2003). Influence of pressure, temperature and surface area/volume ratio on the texture of pyrolytic carbon deposited from methane. Carbon.

[72] Dong G.L., Hüttinger K.J. (2002). Consideration of reaction mechanisms leading to pyrolytic carbon of different textures. Carbon.

[73] De Pauw V., Collin A., Send W., Hawecker J., Gerthsen D., Pfrang A., Schimmel T. (2006). Deposition rates during the early stages of pyrolytic carbon deposition in a hot-wall reactor and the development of texture. Carbon.

[74] Hu Z.J., Hüttinger K.J. (2002). Mechanisms of carbon deposition—A kinetic approach. Carbon.

[75] Vignoles G.L., Langlais F., Descamps C., Mouchon A., Le Poche H., Reuge N., Bertrand N. (2004). CVD and CVI of pyrocarbon from various precursors. Surf. Coatings Technol..

[76] Sadezky A., Muckenhuber H., Grothe H., Niessner R., Pöschl U. (2005). Raman microspectroscopy of soot and related carbonaceous materials: Spectral analysis and structural information. Carbon.

[77] Zambrzycki M., Łoś S., Fraczek-Szczypta A. (2021). Structure and electrical transport properties of carbon nanofibres/carbon nanotubes 3D hierarchical nanocomposites: Impact of the concentration of acetylacetonate catalyst. Ceram. Int..

[78] Zambrzycki M., Jeleń P., Fraczek-Szczypta A. (2022). Structure and electrical transport properties of electrospun carbon nanofibers/carbon nanotubes 3D hierarchical nanocomposites: Effect of the CCVD synthesis conditions. J. Mater. Sci..

[79] Ferrari A.C., Basko D.M. (2013). Raman spectroscopy as a versatile tool for studying the properties of graphene. Nat. Nanotechnol..

[80] Schuepfer D.B., Badaczewski F., Guerra-Castro J.M., Hofmann D.M., Heiliger C., Smarsly B., Klar P.J. (2020). Assessing the structural properties of graphitic and non-graphitic carbons by Raman spectroscopy. Carbon.

[81] Ma B., Rodriguez R.D., Ruban A., Pavlov S., Sheremet E. (2019). The correlation between electrical conductivity and second-order Raman modes of laser-reduced graphene oxide. Phys. Chem. Chem. Phys..

[82] Ferrari A.C. (2007). Raman spectroscopy of graphene and graphite: Disorder, electron–phonon coupling, doping and nonadiabatic effects. Solid State Commun..

[83] Zhou J., Sun G., Zhan Z., An J., Zheng L., Xie E. (2013). Probing structure and strain transfer in dry-spun carbon nanotube fibers by depth-profiled Raman spectroscopy. Appl. Phys. Lett..

[84] Torrisi L., Scolaro C. (2017). Blood Wettability of Haemocompatible Carbon-based Materials. J. Adv. Chem. Eng..

[85] Vigano G., Ten Brink G., Pollack D.K.M., Mariani M.A., Kooi B.J. (2020). Wettability Properties of Standard Pyrolytic Carbon Bileaflet Mechanical Heart Valve Prostheses. Struct. Hear..

[86] Xie J., Xin D., Cao H., Wang C., Zhao Y., Yao L., Ji F., Qiu Y. (2011). Improving carbon fiber adhesion to polyimide with atmospheric pressure plasma treatment. Surf. Coatings Technol..

[87] An F., Lu C., Guo J., He S., Lu H., Yang Y. (2011). Preparation of vertically aligned carbon nanotube arrays grown onto carbon fiber fabric and evaluating its wettability on effect of composite. Appl. Surf. Sci..

[88] Contact Angle of Water on Smooth Surfaces and Wettability. http://www.uskino.com/articleshow_113.html.

[89] Lopez-Suarez L., Al Awabdh S., Coumoul X., Chauvet C. (2022). The SH-SY5Y human neuroblastoma cell line, a relevant in vitro cell model for investigating neurotoxicology in human: Focus on organic pollutants. Neurotoxicology.

[90] Xie H., Hu L., Li G. (2010). SH-SY5Y human neuroblastoma cell line: In vitro cell model of dopaminergic neurons in Parkinson’s disease. Chin. Med. J..

[91] Majhy B., Priyadarshini P., Sen A.K. (2021). Effect of surface energy and roughness on cell adhesion and growth—Facile surface modification for enhanced cell culture. RSC Adv..

[92] Zhu L., Luo D., Liu Y. (2020). Effect of the nano/microscale structure of biomaterial scaffolds on bone regeneration. Int. J. Oral Sci..

[93] Giljean S., Bigerelle M., Anselme K. (2014). Roughness statistical influence on cell adhesion using profilometry and multiscale analysis. Scanning.

[94] Robinson D., Efrat M., Mendes D.G., Halperin N., Nevo Z. (1993). Implants composed of carbon fiber mesh and bone-marrow-derived, chondrocyte-enriched cultures for joint surface reconstruction. Bull. Hosp. Jt. Dis..

[95] Sengupta B., Gregory W.E., Zhu J., Dasetty S., Karakaya M., Brown J.M., Rao A.M., Barrows J.K., Sarupria S., Podila R. (2015). Influence of carbon nanomaterial defects on the formation of protein corona. RSC Adv..

[96] Atilhan M., Costa L.T., Aparicio S. (2019). On the interaction between carbon nanomaterials and lipid biomembranes. J. Mol. Liq..

[97] Baoukina S., Monticelli L., Tieleman D.P. (2013). Interaction of Pristine and Functionalized Carbon Nanotubes with Lipid Membranes. J. Phys. Chem. B.

